# Proficiency and Interlaboratory Variability in the Determination of Phthalate and DINCH Biomarkers in Human Urine: Results from the HBM4EU Project

**DOI:** 10.3390/toxics10020057

**Published:** 2022-01-26

**Authors:** Hans G. J. Mol, Ingrid Elbers, Claudia Pälmke, Daniel Bury, Thomas Göen, Marta Esteban López, Stefanie Nübler, Vincent Vaccher, Jean-Philippe Antignac, Darina Dvořáková, Jana Hajšlová, Amrit Kaur Sakhi, Cathrine Thomsen, Katrin Vorkamp, Argelia Castaño, Holger M. Koch

**Affiliations:** 1 Wageningen Food Safety Research, Part of Wageningen University & Research, 6708 WB Wageningen, The Netherlands; ingrid.elbers@wur.nl; 2 Institute for Prevention and Occupational Medicine of the German Social Accident Insurance, Institute of the Ruhr-University Bochum, 44789 Bochum, Germany; paelmke@ipa-dguv.de (C.P.); bury@ipa-dguv.de (D.B.); koch@ipa-dguv.de (H.M.K.); 3 Institute and Outpatient Clinic of Occupational, Social and Environmental Medicine, Friedrich-Alexander Universität Erlangen-Nürnberg, 91054 Erlangen, Germany; thomas.goeen@fau.de (T.G.); stefanie.nuebler@fau.de (S.N.); 4 National Centre for Environmental Health, Instituto de Salud Carlos III, 28220 Majadahonda, Spain; m.esteban@isciii.es (M.E.L.); castano@isciii.es (A.C.); 5 LABERCA, Oniris, INRAE, 44307 Nantes, France; vincent.vaccher@oniris-nantes.fr (V.V.); jean-philippe.antignac@oniris-nantes.fr (J.-P.A.); 6 Department of Food Analysis and Nutrition, Faculty of Food and Biochemical Technology, University of Chemistry and Technology, 166 28 Prague, Czech Republic; Darina.Dvorakova@vscht.cz (D.D.); Jana.Hajslova@vscht.cz (J.H.); 7 Department of Environmental Health, Norwegian Institute of Public Health, 0213 Oslo, Norway; AmritKaur.Sakhi@fhi.no (A.K.S.); Cathrine.Thomsen@fhi.no (C.T.); 8 Department of Environmental Science, Aarhus University, DK-4000 Roskilde, Denmark; kvo@envs.au.dk

**Keywords:** QA/QC, proficiency testing, human biomonitoring (HBM), interlaboratory comparison investigation (ICI), external quality assurance scheme (EQUAS), HBM4EU

## Abstract

A quality assurance/quality control program was implemented in the framework of the EU project HBM4EU to assess and improve the comparability of biomarker analysis and to build a network of competent laboratories. Four rounds of proficiency tests were organized for 15 phthalate and two DINCH urinary biomarkers (0.2–138 ng/mL) over a period of 18 months, with the involvement of 28 laboratories. A substantial improvement in performance was observed after the first round in particular, and by the end of the program, an average satisfactory performance rate of 90% was achieved. The interlaboratory reproducibility as derived from the participants’ results varied for the various biomarkers and rounds, with an average of 24% for the biomarkers of eight single-isomer phthalates (e.g., DnBP and DEHP) and 43% for the more challenging biomarkers of the mixed-isomer phthalates (DiNP, DiDP) and DINCH. When the reproducibility was based only on the laboratories that consistently achieved a satisfactory performance, this improved to 17% and 26%, respectively, clearly demonstrating the success of the QA/QC efforts. The program thus aided in building capacity and the establishment of a network of competent laboratories able to generate comparable and accurate HBM data for phthalate and DINCH biomarkers in 14 EU countries. In addition, global comparability was ensured by including external expert laboratories.

## 1. Introduction

Phthalates are esters of phthalic acid and mainly used as plasticizers, i.e., substances added to plastics to adjust and improve their material properties. Several phthalates are known endocrine disruptors and reproductive toxicants in animals with adverse effects also assumed for humans [1,2,3]. Apart from their individual toxicity, phthalates are also known to exert their toxicity in combination with other phthalates and with other anti-androgens [4,5]. Currently, in the European Union, 16 phthalates are classified as reproductive toxicants category 1B (EC 1272/2008, Annex VI). Because of their endocrine disrupting properties, 14 of these phthalates have also been classified as Substances of Very High Concern and are included in Annex XIV of the REACH-Directive (EC 1907/2006). Only very few and time limited niche applications have been authorized under REACH so far (e.g., for DEHP and DBP; Table 1), effectively banning these phthalates from use in the EU (details see EC 1907/2006, Annex XVII, entry 51).

Despite these regulatory measures, exposure to phthalates is still ubiquitous in Europe and other parts of the world. However, exposures have shifted considerably in the last two decades, with decreases for the regulated phthalates, recognized as a health concern, and increases for phthalates regarded as less problematic or phthalate substitutes, such as DINCH or DEHTP [6,7,8,9,10,11,12]. Before the turn of the millennium, more than half of the population exceeded the exposure levels deemed acceptable for combined anti-androgenic phthalate exposure (Hazard Index > 1), driven by DBP and DEHP, often exceeding their acceptable individual levels. In the last few years, exceedances for individual phthalates have become rare, and combined/mixed exposures have become of increased concern instead. These ongoing substantial population exposures, especially of children, but also in occupational settings, confirm the need for the continued monitoring of the wide spectrum of phthalate exposures, including their substitutes [13,14,15,16,17].

Human biomonitoring (HBM) of phthalates and their substitutes is regarded as an ideal tool to measure and evaluate all relevant cumulative exposures, because HBM integrates all exposure routes (oral, dermal, inhalation) and all sources, known or unknown, e.g., food, product use, and lifestyle. HBM of phthalates and their alternatives has rapidly progressed over the past two decades, driven by the growing knowledge of the human metabolism, and the increasing availability of appropriate biomarker standards for chemical analyses [18,19,20,21]. The preferred matrix for population HBM of phthalates and their alternatives is urine, and the most appropriate exposure biomarkers are their monoester metabolites, with oxidations of the alkyl side chain becoming more relevant with increasing alkyl chain length [22,23,24,25].

Despite the high number of studies on phthalate HBM, the comparability among them is sometimes limited due to differences in the target populations, biomarkers analyzed, differences in chemical analysis, etc. So far, the only EU project focused on HBM harmonization was COPHES/DEMOCOPHES, but at that time this was only for five phthalates (DEHP, DnBP, DiBP, BBzP, and DEP) [26,27]. Since 2017, the European Human Biomonitoring Initiative HBM4EU (www.hbm4eu.eu accessed 20 January 2022) has continued working on the challenge of achieving coordinated HBM in Europe, and part of these efforts have been focused on the analysis of phthalates. In this regard, an important gap that has been identified is the lack of appropriate proficiency tests in the field of human biomonitoring. At the moment, only one external quality assessment scheme, i.e., G-EQUAS (www.g-equas.de accessed 20 January 2022), exists, including four phthalate biomarkers. Recently, two urine standard reference materials of the National Institute of Standards and Technology (NIST) have become available, providing reference values for 11 biomarkers from six phthalates [28]. Based on the selection of the most relevant phthalates and their biomarkers performed within HBM4EU [25], 15 biomarkers from 10 phthalates complemented by two biomarkers for the substitute DINCH were chosen for the quality assurance/quality control (QA/QC) program of HBM4EU (Table 1). One of the central aims of HBM4EU was to establish and grow a network of competent analytical laboratories across Europe to ensure a broad anchoring of HBM in Europe with high comparability and accuracy of analytical results [29]. Only laboratories that met certain criteria set in the QA/QC program for the respective parameters qualified for chemical analysis within HBM4EU. The principle design and implementation of the laboratory QA/QC program has been described by Esteban López et al. [30]. The current paper presents the specific design and results of the QA/QC program for 17 urinary biomarkers of ten phthalate plasticizers and the phthalate substitute DINCH and discusses challenges and experiences.

## 2. Materials and Methods

### 2.1. Design of the HBM4EU QA/QC Program

The main objectives of the QA/QC program established in HBM4EU were (i) to assess the proficiency of laboratories, (ii) to improve interlaboratory comparability (capacity building and creating a network of competent laboratories), and (iii) to gain insight into the interlaboratory variability. The principle design of the QA/QC program has been described in detail by Esteban López et al. [30], and is briefly summarized here. The proficiency test (PT) scheme consisted of four PT rounds for phthalates/DINCH organized over a period of 18 months (June 2018–December 2019). Each round included two different urine samples containing the biomarkers of interest at levels relevant for HBM (lower and higher concentration range) in the general population. Within HBM4EU, following a literature inventory, it was decided to prioritize 15 phthalate urinary biomarkers, reflecting exposure to 10 phthalates [25]. For DINCH, two urinary biomarkers were included in the scope of analysis (see Table 1). Participation in the QA/QC program was open to laboratories from within the HBM4EU joint effort, covering 30 countries in Europe, and Israel. In total, 35 candidate laboratories (19 countries) expressed their interest in participation for the determination of phthalate biomarkers, and 13 laboratories (10 countries) for DINCH biomarkers.

In the first round, the basic proficiency of the participating laboratories was assessed based on the consensus value derived from all participants’ results according to the principles of an interlaboratory comparison investigation (ICI) (for details see below). In the following rounds, this was changed to a performance assessment against expert values derived from results from three to six expert laboratories, according to the principles of an external quality assessment scheme (EQUAS). The expert laboratories were assigned based on long-term experience regarding the determination of the biomarkers in urine, and a corresponding track-record in peer-reviewed publications. The expert laboratories were not necessarily part of the HBM4EU network. In fact, several external laboratories from the USA were actively approached and kindly contributed.

### 2.2. Preparation and Characterization of Control Materials

For the preparation of the control materials, various archived human urine samples known to contain phthalate and DINCH biomarkers were pooled and mixed. All samples originated from real-life environmental and occupational exposure incidences and thus contained all biomarkers in their metabolism dependent mixture of phase one functionalized and phase two conjugated biomarkers. No external spiking was performed. Target concentrations were chosen to reflect exposure levels in the general adult and child population at roughly the 25th percentile for the low-concentration control materials and between the 75th and 95th percentile for the high-concentration control materials [6,11,15,31,32]. Since native materials were used, covering wide exposure ranges and often complex mixtures of exposures, concentrations could not be fully tailored for each individual biomarker. Biomarkers of less abundant, rarely detected phthalates were therefore often in the lower concentration range or even below method limits of quantification (LOQs) in some of the low-concentration control materials. For pooling, selected materials were thawed, the appropriate volumes taken and mixed. Each pooled control material (approx. 500 mL) was centrifuged to remove any precipitates and ensure homogeneity. Then the material was aliquoted (4 mL portions) into coded polypropylene tubes with a screwcap. The tubes were stored in the freezer (<18 °C). Some of the tubes were stored at −80 °C for future stability testing.

In total, eight control materials were prepared, in sets of two, before the start of each round. For each material, the homogeneity was evaluated following ISO 13528:2015 [33] and Fearn et al. [34], and according to the SOPs elaborated for the HBM4EU QA/QC program. This involved duplicate analysis of 10 randomly selected test samples of each control material, the determination of the average concentration, and between-sample standard deviation. The stability was assessed using the four control materials prepared in rounds 1 and 2, in line with ISO 13528:2015 [33] and the international harmonized protocol for the proficiency testing of analytical laboratories [35]. For this, mean concentrations, which were obtained via the analysis of six test samples for each control material stored in the freezer (<−18 °C) for a time covering at least the period from preparation to PT-reporting due date, were compared, via a *t*-test, with the means of six replicate analyses of the same material stored at −80 °C on the date of preparation of the material.

The analysis method used for homogeneity and stability testing of the phthalate biomarkers has been described in detail before [6]. In brief, 300 µL of the urine sample was mixed with 100 µL ammonium acetate buffer (1 M, pH 6.0–6.4), 10 µL of internal standard solution (isotope labelled analogues), and 6 µL of β-glucuronidase from *E. coli* strain K12. Enzymatic deconjugation was performed for 2.5 h at 37 °C, after which the pH was adjusted by the addition of 10 µL of acetic acid. After a freeze out of proteins and centrifugation, 10 µL of the deconjugated urine was injected into an on-line SPE-HPLC-MS/MS system. For the phthalate biomarkers, the analytes were first trapped on a 10 × 4 mm, 5 µm enrichment column (Capcell Pak^®^C18-MG-II, Phenomenex, Aschaffenburg, Germany) using an eluent composition of 20% acetonitrile/water (0.05% acetic acid). Then, the enrichment column was switched in-line with the analytical column (2.1 × 150 mm; 3 µm, Atlantis dC18, Waters, Eschborn, Germany) and the analytes were eluted and separated using a linear gradient up to 75% acetonitrile/water (0.05% acetic acid). Calibration standards in water were analyzed in the same way. Mass spectrometric detection was performed on an AB Sciex 4500 triple quadrupole mass spectrometer using electrospray in negative ionization mode (ESI^−^). The mass spectrometer was operated in time-programmed multiple reaction monitoring mode. Quantification was based on multi-level calibrations after normalization of the response to the corresponding labelled internal standard for each biomarker. The method LOQs were 0.2 ng/mL for most phthalate biomarkers (0.5 ng/mL for MEP, MBzP, and MnBP; Table 1). DINCH biomarkers were determined in a similar manner to Schütze et al. [21] by a separate injection of 25 µL and a slightly different gradient. The method LOQ was 0.05 ng/mL for each of the DINCH biomarkers.

### 2.3. Organization of Proficiency Tests

Laboratories that had expressed their interest in participating in the HBM4EU QA/QC program received an invitation to register. Test materials (aliquots of 4 mL) were dispatched by courier in frozen conditions in an insulation box with dry ice and usually delivered within 24–48 h. Participants were instructed to analyze the test materials only once, using their own method. They were asked to report the concentration in ng/mL through a web-tool, and, in addition, to provide details of their methods (regarding deconjugation, extraction, instrumental analysis, use of internal standards, method of quantification, and identification parameters) as well as the method LOQ in a fixed format using an excel sheet developed for this purpose. The time for analysis and reporting of results was approximately five weeks.

In total, four rounds were performed between June 2018 and December 2019. Materials were distributed to the participants in June 2018, December 2018, May 2019, and November 2019.

After each round and before the next one, the participants received a feedback report with the results and general recommendations for improving performance. In addition, after the first round, a webinar was organized to discuss the results, possible pitfalls, and to provide suggestions for solving specific difficulties in the analysis.

### 2.4. Assessment of Laboratory Performance

#### 2.4.1. Quantitative Performance

Laboratory performance was assessed by the calculation of z-scores for each biomarker in the test samples. The z-score is a value that relates the reported value to an assigned reference value, taking an estimated feasible and acceptable variability into account, according to the following formula:

(1) $$Z=\frac{x-A}{\sigma_{T}}$$

with Z = z-score

x = participant’s result.

A = assigned value.

σ_T_ = standard deviation for proficiency, with σ_T_ = 0.25 × A

A z-score of |Z| ≤ 2 was interpreted as satisfactory, 2 < |Z| < 3 as questionable, and |Z| ≥ 3 as unsatisfactory in terms of performance. The assigned value was either the consensus value derived from the participants’ results (round 1, ICI) or a reference value based on results from expert laboratories (rounds 2–4, EQUAS). The standard deviation for proficiency (target standard deviation) used was a fixed 25% of the assigned value. Owing to a lack of extensive existing data on achievable and realistic interlaboratory reproducibility (RSD_R_) in HBM analysis, this was considered as initial fit-for-purpose criterion (detailed considerations, see [30]).

In the first round, the consensus value was taken as the assigned value. For the calculation of the consensus value, robust statistics was used (Algorithm A, ISO 13528:2015 [33]). With robust statistics, outliers are not discarded but have only a minor influence on the performance parameters. A minimum of at least seven results and an uncertainty (u) of the consensus value less than 0.7 × σ_T_ were required, with u being 1.25 times the standard deviation of the participants’ results divided by the square root of the number of participants. If these requirements were not met, the data set was considered unfit (too small or with too high a degree of uncertainty) to derive a meaningful consensus value. In such case, no z-scores were calculated. For further details, the reader is referred to [30].

For the second to fourth round, expert values were used as the assigned value. Here, multiple expert laboratories (four to six for phthalates, three for DINCH) analyzed six test samples of each control material in duplicate and reported their results and details of their method to the PT organizer. The use of isotopically labelled analogues of the respective biomarkers as internal standard, added to the urine aliquot before analysis, was a prerequisite. A few incidental non-compliances were observed. In such cases, the results for the respective biomarker/expert laboratory was excluded from the set. For the long-chain mixed-isomer phthalate biomarkers (OH-MiNP, cx-MiNP, OH-MiDP, cx-MiDP) and the two mixed-isomer DINCH biomarkers (OH-MINCH, cx-MINCH), data were only included if prescribed quantifier transitions were used (for explanation, see the Results and Discussion section). For each expert laboratory, the mean value was calculated for each biomarker. Based on these means, the mean of the expert laboratories, the relative standard deviation (RSD), and the relative uncertainty (RSD divided by the square root of the number of expert laboratories) were calculated. The expert value was considered suitable for use as the assigned value as long as the uncertainty did not exceed 0.7 × σ_T_ (i.e., 17.5%) [30]. If it exceeded this criterion, the expert means were checked for outliers using the Grubbs’ test and discarded if identified as such. If the uncertainty still did not meet the criterion of 0.7 × σ_T_, or if the number of remaining expert laboratories was less than three, it was investigated whether a meaningful consensus value could be determined based on the combined results from the expert laboratories and the participants, following the procedure as described above for the first round.

#### 2.4.2. False Negatives and False Positives

For the individual results of the participants, no z-score could be calculated for biomarkers reported as <LOQ. In this case, the reported LOQ was compared with the assigned value. If the participants’ LOQ was below the assigned value, a proxy-z-score was calculated using equation (1) with the reported LOQ as concentration. If the z-score was below −3 (LOQ 4× lower than the assigned value), the result was assigned as a false negative and classified as unsatisfactory since the participant should have been able to detect and quantify the biomarker. For cases in which a participant reported the presence of a biomarker while it was not present in the material (<LOQ of the organizer/expert laboratories, and the majority of the participants), the result was classified as a false positive when the concentration was at least 4× the organizers’ LOQ.

## 3. Results and Discussion

### 3.1. Homogeneity and Stability Testing

For each of the four PT rounds, two control materials were prepared as described in 2.2 and tested for homogeneity prior to shipment to the participants. The mean concentration for all biomarkers in the eight control materials are provided in the supplementary material (Table S1). The lowest concentrations were mostly in the range of 1–2 ng/mL (0.2 ng/mL for MCHP and MnOP, 15 ng/mL for MEP). MCHP and MnPeP were <LOQ in three materials, MEHP in one. The highest concentrations were generally in the range of 10–40 ng/mL, with lower and higher exceptions (1 ng/mL for MCHP, 138 ng/mL for MEP). These concentrations compare well with the range of concentrations (and detection rates) observed in urine samples from adult and child populations in Europe [6,11,27,36,37]. The overall repeatability (RSD_r_) observed during homogeneity testing was generally below 5% (Table S1). The control material was considered sufficiently homogeneous when the between-sample standard deviation did not exceed a critical value (0.3× target standard deviation). This requirement was met in all cases. Example data sheets are included in the supplementary material for illustration (Table S2). In three cases, the within-sample standard deviation was higher than desired for homogeneity assessment but could be explained by the very low MCHP concentration in the R3B and R4A materials and the low MnBP concentration in the R3A material. Given the overall results and the nature of the material (liquid), the control material was nonetheless also considered homogeneous in these cases.

To ensure the concentrations did not change during the period between the preparation of the material and analysis by the participants, aliquots of the material stored in the freezer (<−18 °C) were measured against aliquots of the same materials stored at −80 °C. Under the latter conditions, the biomarkers were considered stable, and this was taken as reference. The storage time covered 96 days for the materials from the first round, and 153 days for the materials from the second round. The difference between the means of results from both storage conditions were generally below 5% and not significant (95% confidence). No instability was observed. Based on the results for these four materials, it was concluded that the control materials were stable for up to at least five months, and further stability testing for the following two rounds was not considered necessary.

### 3.2. Values for the Expert Laboratories

For the second to fourth round, expert values were used as the assigned values. For this, the mean of the concentrations as determined by designated expert laboratories were used, providing they were in reasonable agreement with each other (for details see Section 2.4.1). The number of results used for calculating the mean of experts varied. For phthalates, six expert laboratories were involved in the second round, which decreased to five and four laboratories in the subsequent rounds. Not all expert laboratories included all phthalate biomarkers in their method. Furthermore, in round-2, Grubbs’ outliers were identified for MEP, MBzP, and cx-MiNP, and were discarded. As a result, the number of values for calculating the mean varied per round and per biomarker. For DINCH biomarkers, there were only three expert laboratories involved. An overview of the expert values, the number of results used for the calculation of the mean, and the uncertainty of the means is provided in the supplementary material (Table S3). Expert values with acceptable uncertainties could be derived for almost all biomarkers in the control materials from rounds 2, 3, and 4. The exceptions were MnOP in material R3A, MnPeP in material R2B, and MCHP and OH-MiNP in material R3B. In addition, no expert values could be obtained for MnOP and MnPeP in material R2A because results were only available from two laboratories. In these cases, instead of the expert values, the consensus value was calculated based on the combined set of experts’ and participants’ data. This resulted in a useful consensus value in four cases. For MnPeP (R2A) and MCHP (R3B), the uncertainty was too high, and no assigned value could be established.

### 3.3. Participants’ Scope, LOQs, and Methods

In total, 35 laboratories expressed their interest in participating in the PT for phthalate biomarkers, and 13 for DINCH biomarkers (in most cases the same laboratories). The number of laboratories that registered and submitted results varied from 18 to 25 for phthalate biomarkers, and 11–12 for DINCH biomarkers (numbers include the expert laboratories). Participation was not consistent in each round, i.e., some laboratories participated only once, others in all four rounds, etc. Overall, 28 laboratories submitted results in at least one round for phthalate and/or DINCH biomarkers.

**Figure 1:**
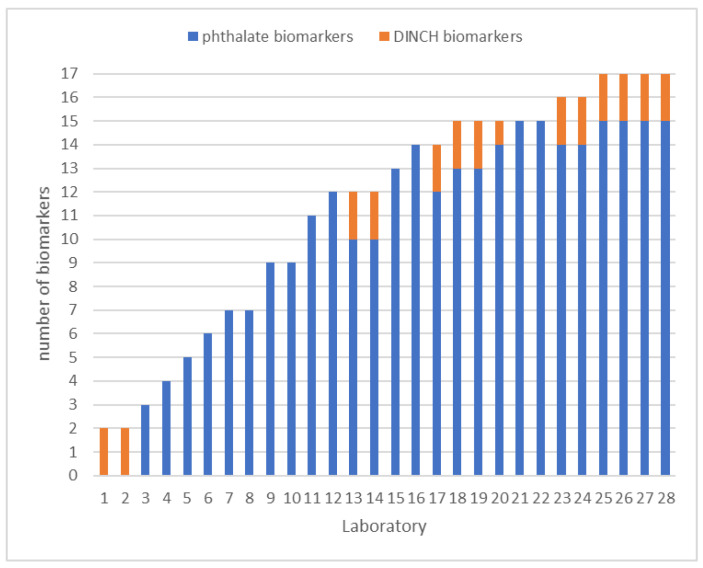
Number of the target phthalate and DINCH biomarkers included in the scope of the laboratories involved in the HBM4EU QA/QC program.

**Figure 2:**
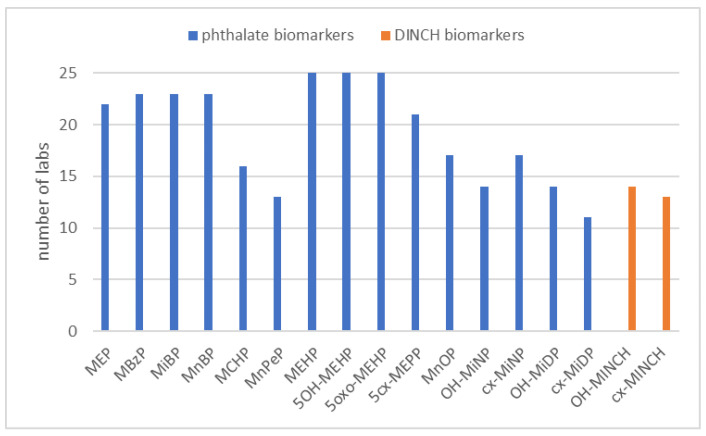
Coverage of each of the target phthalate and DINCH biomarkers by the laboratories involved in the HBM4EU QA/QC program.

Within the frame of HBM4EU, 15 phthalate biomarkers and two DINCH biomarkers were prioritized as target compounds. However, for participation in the HBM4EU QA/QC program, full coverage was not mandatory. The PT showed that the scope of the participants varied greatly, ranging from three phthalate biomarkers or two DINCH biomarkers to all 17 biomarkers (see Figure 1). On average, 12 biomarkers were covered. MEHP, 5OH-MEHP, and 5oxo-MEHP were the ones that were measured by most laboratories. MnPeP and the mixed-isomer phthalate and DINCH biomarkers had the lowest coverage (e.g., only 11 laboratories measured cx-MiDP, see Figure 2).

**Figure 3:**
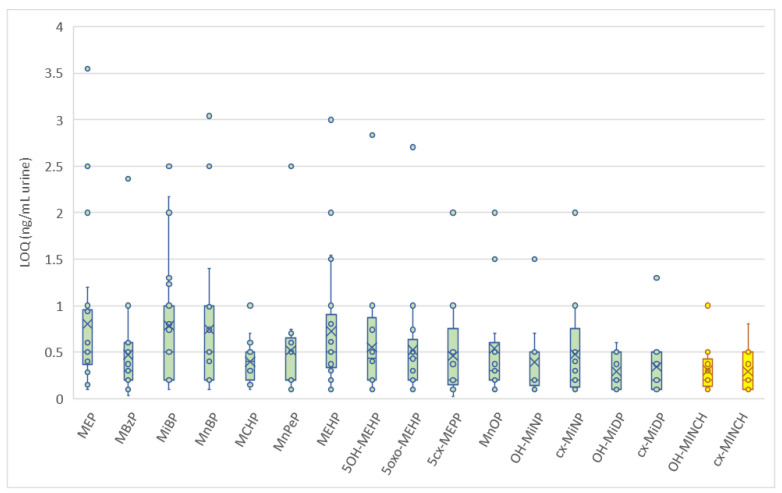
Box–whisker plot of the LOQs reported by the laboratories for each of the target biomarkers in urine.

All laboratories were asked to report their LOQ for each of the biomarkers at the time of registration and also when reporting the results. As for the scope, no minimum required LOQ was specified for participation in the program. It was observed that during reporting and after the first round, several laboratories adjusted their initially stated LOQ. From the second round onwards, the LOQs reported by the laboratories were consistent. These are shown in Figure 3. As can be seen from this figure, the LOQs differed amongst the participants. It was not asked how the LOQs were determined (multiple options exist), so it cannot be excluded that besides the method and instrument used, the procedure chosen for the determination of the LOQ also contributed to these differences. Generally, the LOQs reported were in the range of 0.25–1 ng/mL and thus fit for purpose for the analyses of samples from the general population. For several biomarkers with known omnipresent occurrence, LOQs higher than 1 ng/mL would lead to insufficient detection rates, underestimating the real extent of exposure. The low-concentration control materials made it possible to assess whether the actual LOQs of the laboratories corresponded to their provided LOQs.

Table 2 shows an overview of the method details as provided by the laboratories. Laboratories that analyzed both phthalates and DINCH biomarkers used the same or a similar method in most cases. The most frequently used procedure could be described in a generalized way as follows: isotopically labelled internal standards and buffer were added to 0.5 mL of urine. Then the biomarkers were enzymatically deconjugated at 37 °C for 2 h. The deconjugated biomarkers were extracted using SPE (either online or off-line) and analyzed by LC-MS/MS with ESI^−^, acquiring two mass transitions. Biomarker identification was based on matching retention time and ion ratio using various tolerances. Quantification involved normalization of the responses to the internal standards and was done against calibrants prepared in solvent, or calibrants in either (synthetic) urine or water processed in the same way as the samples (procedural calibration).

### 3.4. Assessment of Laboratory Performance

#### 3.4.1. First Round Experiences

The approach initially chosen to assess the laboratory performance was through z-scores using the participants’ consensus (robust mean) as the assigned value. The first round revealed that variability in results in general was rather high, i.e., exceeding the 25% standard deviation for proficiency (see Section 2.4.1) in most cases (see RSD_R_ in Table 3). For MnPeP, the four mixed isomer phthalate biomarkers, and the two DINCH biomarkers, the variability in results was too high to establish consensus values. Hence, no performance assessment was possible for these biomarkers in the first round. This triggered two actions: (i) the organization of a webinar to discuss possible issues and to provide recommendations to improve comparability, (ii) the switch from using the participants’ consensus as the assigned value to the use of a reference concentration derived from replicate analysis of the control materials by at least three expert laboratories (for details see Section 2.4.1 and Section 3.2).

**Figure 4:**
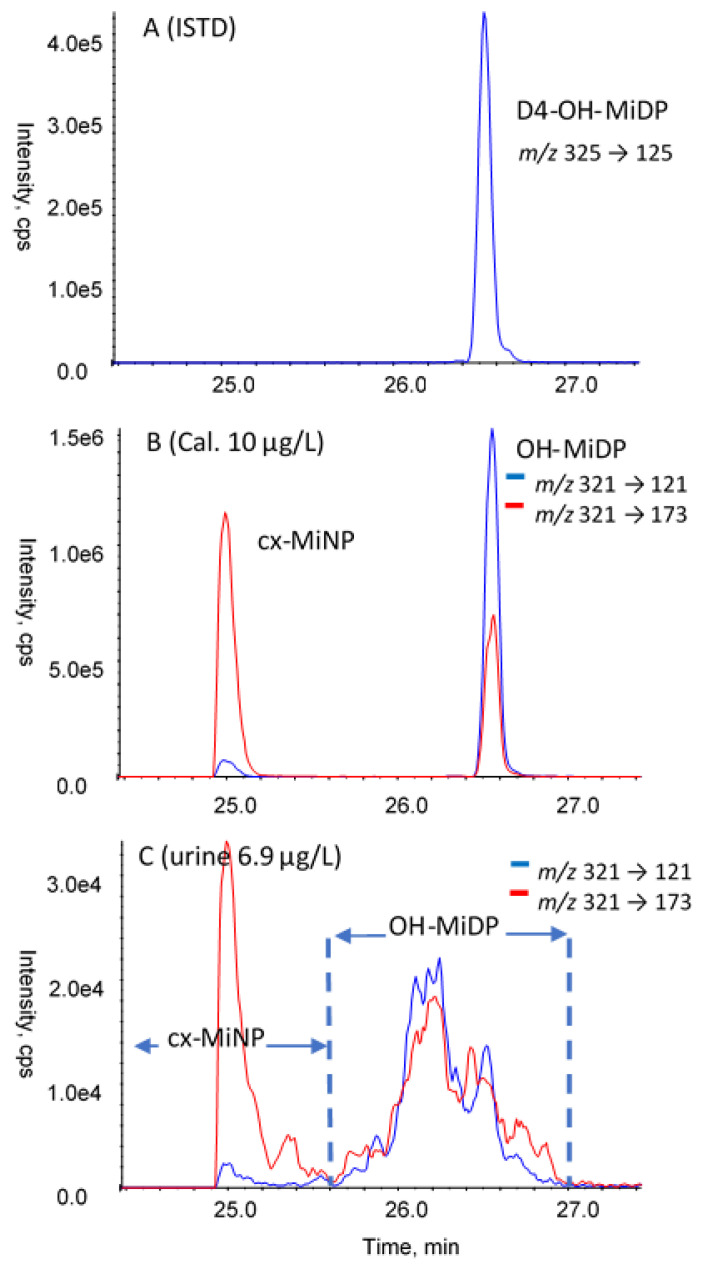
Examples of extracted ion chromatograms for OH-MiDP (quantifier *m/z* 321 > 121, qualifier *m/z* 321 > 173). (**A**) deuterated internal standard; (**B**) calibration standard in solvent (also containing the other phthalate biomarkers); (**C**) urine sample from real-life exposure.

Several pitfalls and potential issues with the determination of phthalate and DINCH biomarkers were identified and discussed during the webinar, and also during training by representatives of expert laboratories in courses and workshops of the HBM4EU training school program:

- Background contamination. In the cases of the monoesters (MEP, MBzP, MnBP, MiBP, and MEHP), external contamination may occur which may cause a positively biased result, especially at the lower concentrations. Careful monitoring by inclusion of multiple procedural blanks can reveal this. If it occurs, the source should be identified and measures taken to prevent background artefacts.
- Peak separation and integration. For MnBP/MiBP, the LC-MS/MS separation is dif-ficult and integration needs attention. For DiNP, DiDP, and DINCH, the biomarkers in real samples originate from isomeric parent compound mixtures. While the analytical (internal) standards yield one defined chromatographic peak, multiple and/or broad peaks are observed in real samples. During the measurement, the acquisition window for these compounds needs to be sufficiently wide to capture the complete mixture. Care needs to be taken during data processing to include all peaks (for example, see Figure 4). Another issue is that for the isomer mixtures, the transition used for quantification affects the quantitative result. For this reason, in the subsequent rounds, the laboratories were instructed to use harmonized quantifier *m/z* transitions (OH-MiNP: 307 > 121; cx MiNP: 321 > 173; OH-MiDP: 321 > 121; cx-MiDP: 335 > 187; OH-MINCH: 313 > 153; cx-MINCH: 327 > 173).
- Internal standard used. All laboratories used internal standards to correct for possible losses or inconsistencies during sample preparation, and to correct for matrix effects in the LC-MS/MS measurement. Especially for the latter, the best option is to use the isotope-labelled analogue for each of the biomarkers analyzed, because matrix effects can be highly variable for the different analytes and the different urine samples. In a substantial number of cases, other isotope-labelled internal standards were used, or even a single isotope-labelled internal standard for all biomarkers analyzed. This may result in sub-optimal or even erroneous correction for matrix effects and deviating analysis results. To illustrate this, the performance obtained with or without using the corresponding isotope-labelled analogue were compared (only results with full details on internal standards used were included). A summary is provided in Table 4. Although satisfactory performance could still be obtained using other internal standards, in all four rounds the results relating to the use of the authentic isotope-labelled analogue were better.
- Enzyme used for deconjugation. Phthalate and DINCH biomarkers in urine of exposed subjects are predominantly present as glucuronides, depending on alkyl chain length and type of oxidative modification [38,39]. HBM analysis is based on the determination of the total aglycone concentration after cleavage of the conjugates. In the cases of phthalates and their substitutes, deconjugation needs to be done carefully because of their labile ester bond(s), which is usually achieved by enzymatic hydrolyses. The type of enzyme, its concentration, pH, and time may affect the resulting concentration of the aglycone. It has been recommended to use pure β-glucuronidase (e.g., from *E. coli* K12) rather than lesser defined or mixed enzyme types such as *Helix Pomatia* β-glucuronidase/aryl sulfatase. While both will result in deglucuronidation, sulfatase/lipase activities present in mixed enzymes from *H. Pomatia* may both cleave the ester-bonds of phthalates (and DINCH) and their biomarkers [40,41]. Thus, early on, phthalate HBM methods were successfully based on ß-glucuronidase-pure enzymes [18,19,40]. However, as indicated in Table 2, roughly a quarter (28%) of the laboratories used enzymes from *H. Pomatia* for deconjugation. It was investigated whether a difference in results could be observed between the laboratories using *E. coli*- and *H. Pomatia*- based enzymes. For this purpose, the data from round-2 were used (highest number of participants). To eliminate bias due to matrix effects in the LC-MS/MS measurement, results were only included when the corresponding isotope-labelled analogue was used as the internal standard in the determination. An additional requirement was that at least three results were available for both groups. A comparison could be made for seven biomarkers in two control materials. The results are included in the supplementary material (Table S5 and Figure S1). For the low-concentration control material R2A, the use of enzymes from *H. Pomatia* resulted in significantly higher concentrations of the simple monoester biomarkers MiBP, MnBP, and MEHP (35%, 49%, and 120%, respectively). This could be explained by the parent diester (that is ubiquitously present) being degraded to the simple monoester, thus artificially elevating their concentrations in the low-concentration control samples. In the high concentration samples, this contribution might be less relevant. In fact, for material R2B, the concentrations reported with *H. Pomatia* appeared slightly lower (less than 20% and therefore not significant), which could be the result of analyte loss through esterase activity in enzyme preparations from *H. Pomatia*. Thus, it seems that the use of *H. Pomatia* results in a positive bias of some biomarkers in the low concentration materials (R2A), and similar results or a negative bias in the high concentration control materials. To summarize, the use of β-glucuronidase pure enzymes is strongly recommended for the determination of phthalate and DINCH biomarkers because: (i) degradation issues related to the arylsulfatase component of mixed enzymes are obvious (resulting in a myriad of quantitatively interfering effects, especially obvious for the monoesters MnBP, MiBP, and MEHP), (ii) human phthalate metabolism data and urinary excretion fractions are based on methods using arylsulfatase-free glucuronidase enzymes, and (iii) most laboratories (including expert laboratories) use these enzymes.

#### 3.4.2. Laboratory Performances along the HBM4EU QA/QC Program

For the second to fourth round, mean concentrations from up to six expert laboratories were used as the assigned value instead of the consensus value. The advantage of this was that performance assessment was also possible in cases where no consensus values could be obtained based on the participants’ results due to either too high a dispersion of results or too low a number of participants for a particular biomarker. Furthermore, the inclusion of highly experienced and worldwide renowned laboratories (also outside of HBM4EU) as expert laboratories increased confidence in the accuracy of the assigned value and data comparability beyond Europe. Nevertheless, consensus values were also calculated in these rounds to compare them with the expert values. The difference between the consensus value and the mean expert value (in % relative to the expert value) is included in Table 3. In general, both values were in good agreement (<20% difference) indicating that the approach was appropriate despite the relatively small number of expert laboratories for some of the biomarkers. A high deviation was only observed for MEHP (material R2A), where the participants’ consensus value was almost twice the expert value. A possible explanation for this could be a lower level of control regarding background contamination amongst some of the participants compared to the expert laboratories, or the use of a mixed arylsulfatase/β-glucuronidase enzyme instead of pure β-glucuronidase.

In Table 3, the percentage of laboratories that obtained satisfactory, questionable, and unsatisfactory performances is shown for each biomarker and each of the four rounds. In this table, the incidence of false negatives and false positives is also indicated. In total, eight false negatives were observed. They concerned assigned concentrations of 1–2 ng/mL and, in two incidences, higher concentrations (5.4 and 12.6 ng/mL). False positives were only observed in the first round, two for MCHP and two for MnPeP.

**Figure 5:**
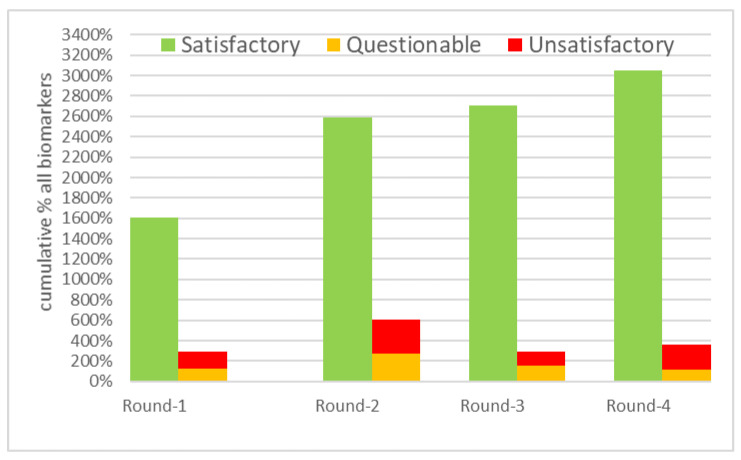
Overall laboratory performance for each round, summing the percentage of satisfactory, questionable, or unsatisfactory z-scores for the 17 biomarkers in the two control materials from each round. The maximum cumulative percentage in a round = 3400% (17 biomarkers × 2 materials × 100%). A sum of satisfactory and questionable/unsatisfactory below 3400% means that performance assessment was not possible for certain biomarkers in the control materials from that round.

In Figure 5, the percentages of laboratories with a satisfactory, questionable, and unsatisfactory z-score for each round were summed for all 17 biomarkers in the two control materials. This cumulative percentage could reach a maximum of 3400% (17 × 2 × 100%) if performance assessment was possible for all biomarkers in both control materials from a round. The cumulative percentage is indicative for the progress made by the laboratories over the 18 months of the HBM4EU QA/QC program. More laboratories achieved satisfactory results with each round. The figure is indicative because the population of laboratories and the control materials differed in each round. The major improvement after the first round was partly due to the capacity building webinar and the switch to expert values as assigned values (i.e., fewer issues with obtaining an assigned value).

As can be seen from Table 3, there are differences in terms of performance at an individual biomarker/material level. The mixed-isomer phthalate and DINCH biomarkers, in addition to MnPeP, were the more challenging ones. z-scores could not always be established, and lower percentages of satisfactory z-scores were obtained, especially in the first rounds. On the other hand, very good performances were seen for 5OH-MEHP, 5oxo-MEHP, and the simple monoesters (after pitfalls, such as external contamination, had been resolved). These were the more commonly analyzed biomarkers (and hence the participants can be assumed to be more experienced), and they also had the highest percentage of participants using the isotope-labelled analogues as internal standards.

### 3.5. Interlaboratory Variability

**Figure 6:**
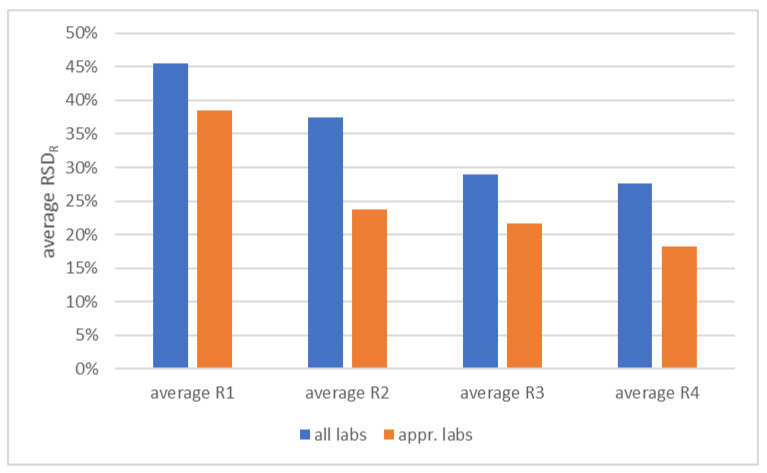
Average RSD_R_ (interlaboratory reproducibility) based on all phthalate and DINCH biomarkers for each of the four rounds. Results shown for two groups of laboratories: all participants and laboratories approved in the framework of the HBM4EU project.

The primary aim of the HBM4EU QA/QC program was to assess and improve the performance of laboratories involved in the determination of prioritized biomarkers of exposure, and to create a network of European laboratories that can generate comparable analytical data [30]. Laboratories yielding satisfactory results for a certain biomarker in both control materials in at least two rounds in the program were considered to generate reliable data for that biomarker (referred to as ‘approved’ laboratories in the framework of HBM4EU). The program was also very valuable in terms of gaining insight into actual interlaboratory variability (RSD_R_) in phthalate and DINCH biomarker analysis, how this developed during the program, and in terms of comparing the variability of the ‘approved’ laboratories to that of all the participants. Figure 6 shows the average RSD_R_ based on all phthalate and DINCH biomarkers for each of the four rounds. This is shown for two groups of laboratories: all participating laboratories, and the laboratories that, based on the results after the fourth round, were approved in the framework of the HBM4EU project. A clear reduction of the average RSD_R_ can be observed after the first round, not only across all participants, but also for the group of approved laboratories. From the second round onwards, further improvements were observed, leveling off towards the fourth round.

**Figure 7:**
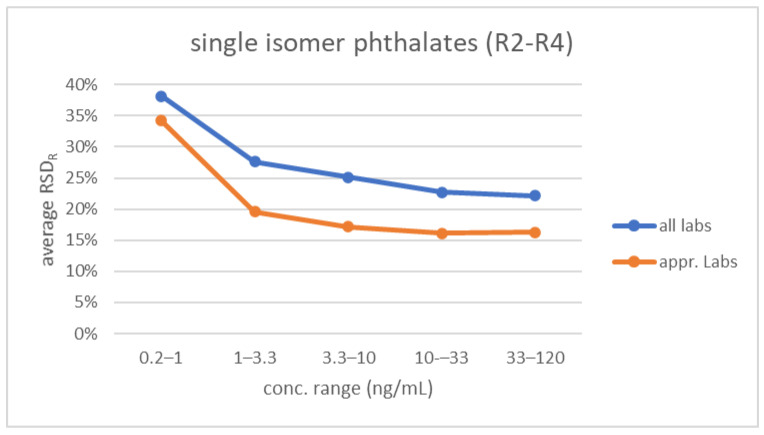

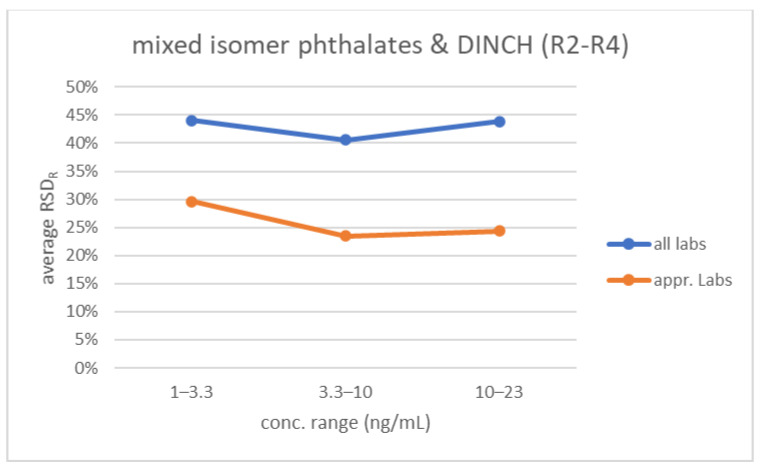
Average RSD_R_ (interlaboratory reproducibility) from rounds 2–4 versus concentration range of the biomarkers in urine. Top: average of 11 single isomer phthalate biomarkers. Bottom: average of four mixed isomer phthalates and two DINCH biomarkers. Results shown for two groups of laboratories: all participants and laboratories approved in the framework of the HBM4EU project. Note: each average reflects a wide range of RSD_R_s (see Figure S3), and not all averages are significantly different from each other (see text).

As discussed previously [30], there is some ambiguity regarding the relationship between RSD_R_ and the concentration of analytes, i.e., whether an increase of RSD_R_ with decreasing analyte concentration was broadly observed for the spectrum of priority compounds included in the HBM4EU QA/QC program. This was investigated here for the phthalate and DINCH biomarkers. Given the pronounced decrease in RSD_R_ after the first round (achieved by providing feedback and harmonization), it was decided that the data from the first round should be excluded, and that the data from rounds 2–4 only should be used for this analysis. Furthermore, because of the higher complexity involved in determining mixed-isomer phthalates and DINCH biomarkers, the assessment for these biomarkers was done separately. The RSD_R_s for the individual biomarkers from both groups are shown in the supplementary material Figure S3. The RSD_R_s are highly scattered, without an obvious trend. To simplify visualization, RSD_R_s were averaged over concentration ranges. The results are depicted in Figure 7.

For the single-isomer phthalate biomarkers, the highest average RSD_R_ (38% when including data for all laboratories) is seen at the lowest concentration range (< 0.2–1 ng/mL, very close to the LOQ of most laboratories). This decreases substantially for the 1–3.3 ng/mL range, and then only slightly decreases further to 22% for the higher concentration ranges. Due to the high variability of the individual RSD_R_s, the difference was significant only for the difference between the average of 0.2–1 ng/mL and the averages of the ranges above 3.3 ng/mL (*p* ≤ 0.05). Between 1 to 120 ng/mL, the differences of the average RSD_R_s were not significant. Thus, it can be concluded that except when being very close to the LOQ (1–3×), the average RSD_R_ is not concentration dependent, and that for the entire group of laboratories, the reproducibility is around 24%. For the mixed-isomer phthalate and DINCH biomarkers, the average RSD_R_s between 1 and 23 ng/mL were a constant 41–44%, thus higher than for the single-isomer phthalate biomarkers. This elevated level of RSD_R_ reflects the higher complexity of the chemical analysis and quantification, due to the presence of multiple isomers to be integrated (see also Figure 4).

Figure 7 also shows the difference between the variability of results generated by the laboratories performing satisfactorily in at least two rounds (‘approved’ laboratories) compared to all participants. For the single isomer phthalate biomarkers, lower average RSD_R_s (16–20%) were obtained, although, again due to the scatter of individual RSD_R_s, the difference was only significant for the range 1–3.3 ng/mL. For the mixed-isomer phthalates/DINCH, the improvement to 23–30% was clearer and significant from 3.3 ng/mL onwards.

Based on the above results, it can also be concluded that, in retrospect, the 25% fixed relative standard deviation for proficiency was overall realistic and appropriate for phthalate/DINCH biomarker analysis.

## 4. Conclusions

A QA/QC program in the framework of HBM4EU was performed for 15 phthalate and two DINCH biomarkers in urine which provided insights into the capabilities of 25 laboratories in Europe. At an individual biomarker level, differences were observed in terms of coverage and performance. The biomarkers most frequently analyzed, and also those with the highest satisfactory performance scores, included the multiple biomarkers of DEHP and the classical monoester biomarkers MBzP, MiBP, and MnBP. MCHP, MnPeP, the mixed isomer phthalates (DiNP, DiDP), and DINCH biomarkers were included less frequently. The former have been introduced rather recently as biomarkers of toxicologically relevant phthalates, while the latter are much more challenging from an analytical perspective and in terms of their limited standard availability.

The first proficiency test round revealed a rather high dispersion of results for several biomarkers and the need for harmonization of quantifier transitions and integration windows for mixed isomer phthalate and DINCH biomarkers. This was expected, however, because participation was open to all laboratories, and therefore the laboratories had various degrees of experience with human biomonitoring of plasticizers. In this regard, the webinar offered after round one to discuss the results, possible pitfalls, and solutions proved particularly helpful. Further and specific training was provided by representatives of the expert laboratories in targeted courses and workshops as part of the HBM4EU training school program. This fruitful collaboration among experts and less skilled laboratories underlines the need for continuous work on the European network of HBM laboratories.

In the subsequent rounds, a substantial improvement in the satisfactory performance of the participants was achieved, and so was an overall reduction in interlaboratory variability. On average, the RSD_R_ was 24% for the single-isomer phthalate biomarkers and 43% for the mixed-isomer phthalate/DINCH biomarkers. For laboratories with consistently good performance in the proficiency tests, this decreased to 17% and 26%, respectively. The inclusion of external international expert laboratories ensured the comparability of the data on a scale beyond Europe. With this, the program succeeded in its aim of building capacity and establishing a network of laboratories with consistent satisfactory performance, thereby contributing to better and more comparable HBM data generated within the HBM4EU project, and beyond.

In order to maintain and further extend the network of competent laboratories, it is strongly recommended that a sustainable QA/QC program offering at least one annual PT for the current scope of prioritized biomarkers is established. For future proficiency testing, a 25% fixed relative standard deviation for proficiency is considered an appropriate benchmark for the determination of phthalate/DINCH biomarkers in urine.

## Figures and Tables

**Table 1:** Biomarkers for phthalates and DINCH included in the HBM4EU QA/QC program and abbreviations used.

Parent Compound	Biomarker(s)	Abbreviation
**Phthalates**		
Diethyl phthalate (DEP)	Mono-ethyl phthalate	MEP
Butyl benzyl phthalate (BBzP)	Mono-benzyl phthalate	MBzP
Di-isobutyl phthalate (DiBP)	Mono-isobutyl phthalate	MiBP
Di-n-butyl phthalate (DnBP)	Mono-n-butyl phthalate	MnBP
Dicyclo-hexyl phthalate (DCHP)	Mono-cyclo-hexyl phthalate	MCHP
Di-n-pentyl phthalate (DnPeP)	Mono-n-pentyl phthalate	MnPeP
Di(2-ethylhexyl)phthalate (DEHP)	Mono(2-ethylhexyl) phthalate	MEHP
	Mono(2-ethyl-5-hydroxy-hexyl) phthalate	5OH-MEHP
	Mono(2-ethyl-5-oxo-hexyl) phthalate	5oxo-MEHP
	Mono(2-ethyl-5-carboxy-pentyl) phthalate	5cx-MEPP
Di-n-octyl phthalate (DnOP)	Mono-n-octyl phthalate	MnOP
Di-isononyl phthalate (DiNP)	7-OH-(mono-methyl-octyl) phthalate	OH-MiNP
	7-Carboxy-(mono-methyl-heptyl) phthalate	cx-MiNP
Di-isodecyl phthalate (DiDP) ^1^	6-OH-Mono-propyl-heptyl phthalate	OH-MiDP
	Mono(2,7-methyl-7-carboxy-heptyl) phthalate	cx-MiDP
**Alternatives: DINCH**		
Di-isononyl cyclohexane-1,2-dicarboxylate (DINCH)	cyclohexane-1,2-dicarboxylate-mono-(7-hydroxy-4-methyl)octyl ester	OH-MINCH
	cyclohexane-1,2-dicarboxylate-mono-(7-carboxylate-4-methyl)heptyl ester	cx-MINCH

^1^ all C10 phthalates including di(2-propylheptyl) phthalate (DPHP).

**Table 2:** Overview of methods used by the laboratories for determination of phthalate and DINCH biomarkers in urine.

Step	Details and Usage by the Laboratories
Pretreatment of urine	none (92%) centrifugation (8%)
Urine aliquot used	0.1–3.0 mL, median 0.5 mL
pH adjustment before deconj.	buffer added, in most cases Na/NH_4_ acetate buffer, pH 6.5
Deconjugation	enzymatic in all cases: *E. coli*-based β-glucuronidase, 37 °C, 0.5–15 h, median 2 h (72%) *Helix Pomatia*-based (β-glucuronidase/sulfatase), 37 °C, 1–2 h or overnight (28%)
Sample adjustment before extraction	acidification with formic acid or acetic acid (67%) none (33%)
Extraction/cleanup	SPE online (40%), SPE off-line (28%), LLE (8%) none/dilute and shoot (24%)
Instrumental analysis	LC-MS/MS (ESI negative mode) (96%) GC-MS after derivatization (4%)
Internal standards used	corresponding isotope-labelled analogue for each biomarker (54%) isotope-labelled biomarker, partially corresponding/partially not (27%) isotope labels used not specified at individual biomarker level (12%) no information provided (8%)
Moment of addition of internal standard to sample	before deconjugation (96%) before extraction (4%)
Quantification	response normalized to internal standard (100%) calibration standards prepared in solvent/eluent (68%) procedural calibration using synthetic urine/blank urine/water (28%) standard prepared in final extract (4%)
**Identification**	
Retention time tolerance used	absolute: <±0.1 min (36%), ±0.1 min (28%), ±0.2–0.5 min (12%) relative, ±0.1−2.5% of ret. time (24%) not specified (8%)
Number of transitions acquired	1 (24%), 2 (68%), 3 (8%)
Ion ratio tolerance used	±15% (13%), ±20% (56%), ±30% (31%)

**Table 3:** Summary of results obtained in the HBM4EU QA/QC program for phthalates and DINCH biomarkers.

Biomarker	R	CM	C(E) ng/mL	C(C) ng/mL	RSD_R_	Δ C(C) Vs. C(E)	N (q) (<LOQ)	S	Q	US
MEP	1	A	^1^	127	28%	-	14	86%	7%	7%
		B	^1^	123	42%	-	14	79%	7%	14%
	2	A	17.4	19.7	27%	13%	21	86%	5%	10%
		B	52.3	59.7	21%	14%	21	81%	10%	10%
	3	A	71.8	77.5	17%	8%	20	85%	5%	10%
		B	103	108	17%	4%	20	95%	0%	5%
	4	A	51.5	56.2	18%	9%	15	93%	0%	7%
		B	122	128	22%	5%	15	93%	0%	7%
MBzP	1	A	^1^	2.60	23%	-	16	88%	0%	13%
		B	^1^	3.96	26%	-	16	94%	6%	0%
	2	A	0.955	1.15	27%	20%	19 (2×<)	76%	5%	19%
		B	10.4	10.4	23%	1%	21	95%	0%	5%
	3	A	<0.2	0.24	29%	-	9 (11×<)	^4^	^4^	^4^
		B	3.21	2.86	19%	−11%	20	90%	10%	0%
	4	A	2.03	2.13	13%	5%	14	93%	0%	7%
		B	2.81	2.98	8%	6%	14	93%	0%	7%
MiBP	1	A	^1^	7.26	32%	-	16	94%	0%	6%
		B	^1^	19.3	30%	-	16	81%	19%	0%
	2	A	8.59	8.48	30%	−1%	21	90%	5%	5%
		B	69.9	65.4	36%	−6%	21	86%	10%	5%
	3	A	1.28	1.45	39%	13%	16 (4×<, 2 FN)	82%	6%	12%
		B	15.3	15.1	19%	−1%	20	95%	0%	5%
	4	A	17.4	17.7	24%	2%	16	100%	0%	0%
		B	17.2	17.8	24%	4%	16	94%	6%	0%
MnBP	1	A	^1^	11.1	26%	-	16	88%	0%	13%
		B	^1^	16.4	31%	-	16	88%	6%	6%
	2	A	6.64	7.63	29%	15%	21	86%	5%	10%
		B	53.9	54.9	25%	2%	21	95%	5%	0%
	3	A	1.03	1.13	34%	10%	18 (2×<)	89%	6%	6%
		B	11.8	11.1	14%	−6%	20	95%	0%	5%
	4	A	13.9	14.2	20%	2%	16	94%	6%	0%
		B	11.0	11.3	25%	3%	16	94%	6%	0%
MCHP	1	A	(<0.2) ^1,6^	^4^			2 + 2 FP (7×<)	^4^	^4^	^4^
		B	^1^	0.925	39%	-	10 (1×<)	90%	10%	0%
	2	A	<0.20	^4,8^			6 (7×<)	^4,8^	^4,8^	^4,8^
		B	1.26	1.43	33%	13%	13	92%	8%	0%
	3	A	< 0.2	^4^			1 (12×<)	^4^	^4^	^4^
		B	(0.29) ^2,6^	^5^	54%		11 (2×<)	^7^	^7^	^7^
	4	A	0.295	0.345	20%	17%	10 (3×<)	90%	0%	10%
		B	0.533	0.625	24%	17%	10 (3×<)	90%	0%	10%
MnPeP	1	A	(<0.2) ^1,6^	^4^			2 FP (7×<)	^4^	^4^	^4^
		B	(1.43) ^1,6^	^5^	59%	-	8 (1×<)	^7^	^7^	^7^
	2	A	(<0.2) ^2,6^	^4^			10 (2×<)	^4^	^4^	^4^
		B	(11.8) ^3^	10.4	49%	−12%	12	75%	25%	0%
	3	A	<0.2	^4^			0 (11×<)	^4^	^4^	^4^
		B	1.32	^5^	43%	-	10 (1×<)	80%	10%	10%
	4	A	1.75	2.33	36%	34%	11 (1×<)	64%	18%	18%
		B	2.50	3.06	19%	22%	12	83%	0%	17%
MEHP	1	A	^1^	1.83	40%	-	16 (2×<)	75%	6%	19%
		B	^1^	8.58	34%	-	18	83%	11%	6%
	2	A	0.567	1.12	51%	98%	17 (6×<)	52%	4%	43%
		B	5.89	6.75	28%	15%	23	87%	4%	9%
	3	A	1.21	1.30	27%	7%	20	90%	5%	5%
		B	4.76	5.11	22%	7%	20	95%	5%	0%
	4	A	3.33	4.01	28%	21%	17	82%	12%	6%
		B	3.98	4.81	25%	21%	17	88%	6%	6%
5OH-MEHP	1	A	^1^	11.3	26%	-	18	89%	0%	11%
		B	^1^	40.1	24%	-	18	78%	6%	17%
	2	A	4.12	4.12	21%	0%	23	100%	0%	0%
		B	32.3	27.6	28%	−14%	23	91%	9%	0%
	3	A	3.01	2.96	21%	−2%	21	95%	5%	0%
		B	27.1	25.4	14%	−6%	21	100%	0%	0%
	4	A	13.2	13.4	13%	2%	17	100%	0%	0%
		B	23.2	23.9	13%	3%	17	94%	0%	6%
5oxo-MEHP	1	A	^1^	5.30	25%	-	18	89%	6%	6%
		B	^1^	18.6	38%	-	18	83%	17%	0%
	2	A	1.74	1.69	18%	−2%	22 (1×<)	91%	4%	4%
		B	14.6	12.5	18%	−14%	23	87%	13%	0%
	3	A	1.44	1.45	18%	1%	20 (1×<)	95%	0%	5%
		B	12.9	12.4	14%	−3%	21	95%	5%	0%
	4	A	5.82	5.85	20%	0%	17	100%	0%	0%
		B	11.1	11.7	12%	5%	17	100%	0%	0%
5cx-MEPP	1	A	^1^	9.62	25%	-	14	93%	7%	0%
		B	^1^	35.6	40%	-	14	71%	21%	7%
	2	A	5.41	4.77	22%	−12%	19 (1×<,1 FN)	85%	5%	10%
		B	33.0	29.7	21%	−10%	20	85%	5%	10%
	3	A	3.22	2.55	36%	−21%	19	89%	0%	11%
		B	28.4	24.0	34%	−15%	19	95%	5%	0%
	4	A	15.6	14.8	29%	−5%	15	93%	7%	0%
		B	24.7	23.3	30%	−6%	15	93%	7%	0%
MnOP	1	A	^1^	1.27	19%	-	10 (1×<)	80%	0%	20%
		B	^1^	6.13	17%	-	11	82%	0%	18%
	2	A	(0.179) ^3^	0.194	34%	8%	10 (4×<)	64%	14%	21%
		B	1.70	2.05	24%	21%	13 (1×<, 1 FN)	79%	14%	7%
	3	A	(0.402) ^3^	^5^	56%	-	10 (4×<)	80%	0%	20%
		B	2.94	3.04	30%	3%	14	79%	7%	14%
	4	A	1.36	1.32	28%	−3%	14 (2×<)	92%	8%	0%
		B	2.57	2.65	39%	3%	14	93%	0%	7%
OH-MiNP	1	A	(7.46) ^1,6^	^5^	82%	-	8	^7^	^7^	^7^
		B	(17.5) ^1,6^	^5^	93%	-	8	^7^	^7^	^7^
	2	A	1.81	1.77	18%	−2%	11 (2×<, 1 FN)	85%	8%	8%
		B	11.2	^5^	57%	-	13	85%	8%	8%
	3	A	1.07	1.35	29%	25%	11 (3×<, 1 FN)	67%	25%	8%
		B	(13.2) ^3^	13.9	26%	5%	14	79%	7%	14%
	4	A	5.80	^5^	51%	-	11	82%	0%	18%
		B	8.17	8.99	27%	10%	11	82%	0%	18%
cx-MiNP	1	A	(7.35) ^1,6^	^5^	69%	-	10 (1×<)	^7^	^7^	^7^
		B	26.3) ^1,6^	^5^	70%	-	10 (1×<)	^7^	^7^	^7^
	2	A	2.64	^5^	50%	-	16 (1×<, 1 FN)	53%	24%	24%
		B	12.6	7.17	39%	−43%	16 (1×<, 1 FN)	63%	19%	19%
	3	A	2.04	2.11	47%	3%	17	82%	12%	6%
		B	19.2	16.7	35%	−13%	17	88%	12%	0%
	4	A	9.25	7.19	34%	−22%	14	86%	7%	7%
		B	15.7	12.8	30%	−19%	14	86%	0%	14%
OH-MiDP	1	A	(6.90) ^1,6^	^5^	98%	-	10	^7^	^7^	^7^
		B	(32.0) ^1,6^	^5^	101%	-	10	^7^	^7^	^7^
	2	A	2.88	^5^	85%		13 (1×<, 1 FN)	57%	14%	29%
		B	17.2	^5^	69%		12 (2×<, 2 FN)	71%	7%	21%
	3	A	1.55	1.65	29%	6%	15	100%	0%	0%
		B	19.1	17.7	27%	−8%	15	100%	0%	0%
	4	A	9.87	^5^	61%		12	83%	0%	17%
		B	15.9	^5^	73%		12	75%	8%	17%
cx-MiDP	1	A	(5.28) ^1,6^	^8^			2 (2×<)	^8^	^8^	^8^
		B	(23.9) ^1,6^	^8^			2 (2×<)	^8^	^8^	^8^
	2	A	1.95	^8^			10	80%	0%	20%
		B	10.0	^8^			10	90%	0%	10%
	3	A	1.80	^8^			10 (1×<, 1 FN)	91%	0%	11%
		B	14.6	^8^			11	100%	0%	0%
	4	A	7.19	^8^			8	88%	13%	0%
		B	13.5	^8^			8	88%	0%	13%
OH-MINCH	1	A	(3.28) ^1,6^	^5^	54%		11	^7^	^7^	^7^
		B	(19.1) ^1,6^	^5^	46%		11	^7^	^7^	^7^
	2	A	6.91	^5^	51%		12	75%	17%	8%
		B	22.9	^5^	49%		12	83%	8%	8%
	3	A	1.09	0.953	41%	−13%	12	83%	17%	0%
		B	13.0	10.7	19%	−18%	12	100%	0%	0%
	4	A	12.3	9.69	32%	−21%	11	91%	0%	9%
		B	9.71	7.91	31%	−19%	11	91%	0%	9%
cx-MINCH	1	A	(3.16) ^1,6^	^5^	70%		10	^7^	^7^	^7^
		B	(14.6) ^1,6^	^5^	57%		10	^7^	^7^	^7^
	2	A	3.67	^5^	55%		11	82%	9%	9%
		B	12.1	^5^	70%		11	82%	9%	9%
	3	A	1.09	^5^	53%		10	80%	10%	10%
		B	8.30	5.04	15%	−39%	10	100%	0%	0%
	4	A	7.07	^8^			9	89%	11%	0%
		B	7.70	^8^			9	89%	0%	11%

R = round. CM = control material. C(E) = expert value (for uncertainty see Table S3), used as assigned value in Rounds 2–4. C(C) = consensus value: robust mean of participants’ results (results expert laboratories not included), used as assigned value in Round-1. RSD_R_ = robust standard deviation based on participants’ results. Δ C(C) vs. C(E) = difference between robust mean and expert value (in % relative to the expert value). N(q) = number of laboratories providing quantitative results, between brackets: number of laboratories reporting “<LOQ” and false positives (FP). The sum of both is the total number of laboratories (including expert laboratories) measuring the biomarker. #FN = number of laboratories reporting <LOQ that was identified as false negative. S = % laboratories with satisfactory performance; Q = % laboratories with questionable performance; US = % laboratories with unsatisfactory performance. ^1^ In the first round, no expert values were established. ^2^ No expert value could be established (too high variability or less than three results). ^3^ No expert value, instead the robust mean of results from participants and expert laboratories was used as the assigned value (details see Section 3.2). ^4^ No assigned value and no z-scores due to the biomarker being below <LOQ of organizer (round-1) or expert laboratories (rounds 2–4). ^5^ Uncertainty too high to establish consensus value based on the participants’ results. ^6^ Concentration as established during homogeneity assessment. ^7^ No z-score because no assigned value could be established. ^8^ No statistics performed because number of participants (excluding expert laboratories) was too low.

**Table 4:** Effect of internal standard used on performance.

		Performance		
	N	Satisfactory	Questionable	Unsatisfactory
R1 using corresponding analogue	193	90%	4%	6%
R1 using other isotope label	77	70%	13%	17%
R2 using corresponding analogue	341	87%	6%	7%
R2 using other isotope label	107	67%	16%	17%
R3 using corresponding analogue	278	92%	5%	3%
R3 using other isotope label	66	86%	9%	5%
R4 using corresponding analogue	242	95%	2%	2%
R4 using other isotope label	70	71%	10%	19%

N = number of individual results (z-scores) included in the comparison. Results were only included when full details on the internal standards used were provided by the laboratory.

## Supplementary Materials

The following supporting information can be downloaded at: https://www.mdpi.com/article/10.3390/toxics10020057/s1, Table S1: Mean biomarkers concentration as determined during homogeneity assessment, Table S2: Example sheets homogeneity assessment, Table S3. Expert values used as assigned values in performance assessment rounds 2–4, Table S4: List of expert laboratories in phthalate and DINCH biomarker QA/QC program, Table S5: Effect enzyme used for deconjugation on results of selected biomarkers, Figure S1: Box Whisker plots effect enzyme used for deconjugation on results of selected biomarkers, Figure S2: Interlaboratory variability of determination of phthalate and DINCH biomarkers in urine, Figure S3: RSD_R_ (interlaboratory reproducibility) from rounds 2–4 versus concentration range of the biomarkers in urine.

## References

[1] Fisher J.S. (2004). Environmental anti-androgens and male reproductive health: Focus on phthalates and testicular dysgenesis syndrome. Reproduction.

[2] Furr J.R., Lambright C.S., Wilson V.S., Foster P.M., Gray L.E. (2014). A Short-term In Vivo Screen Using Fetal Testosterone Production, a Key Event in the Phthalate Adverse Outcome Pathway, to Predict Disruption of Sexual Differentiation. Toxicol. Sci..

[3] Lioy P.J., Hauser R., Gennings C., Koch H.M., Mirkes P.E., Schwetz B.A., Kortenkamp A. (2015). Assessment of phthalates/phthalate alternatives in children’s toys and childcare articles: Review of the report including conclusions and recommendation of the Chronic Hazard Advisory Panel of the Consumer Product Safety Commission. J. Exp. Sci. Environ. Epidemiol..

[4] Howdeshell K.L., Hotchkiss A.K., Earl Gray L. (2017). Cumulative effects of antiandrogenic chemical mixtures and their relevance to human health risk assessment. Int. J. Hyg. Environ. Health.

[5] Kortenkamp A., Koch H.M. (2020). Refined reference doses and new procedures for phthalate mixture risk assessment focused on male developmental toxicity. Int. J. Hyg. Environ. Health.

[6] Koch H.M., Rüther M., Schütze A., Conrad A., Pälmke C., Apel P., Brüning T., Kolossa-Gehring M. (2017). Phthalate metabolites in 24-h urine samples of the German Environmental Specimen Bank (ESB) from 1988 to 2015 and a comparison with US NHANES data from 1999 to 2012. Int. J. Hyg. Environ. Health.

[7] Zota A.R., Calafat A.M., Woodruff T.J. (2014). Temporal trends in phthalate exposures: Findings from the National Health and Nutrition Examination Survey, 2001–2010. Environ. Health Perspect..

[8] Kasper-Sonnenberg M., Koch H.M., Apel P., Rüther M., Pälmke C., Brüning T., Kolossa-Gehring M. (2019). Time trend of exposure to the phthalate plasticizer substitute DINCH in Germany from 1999 to 2017: Biomonitoring data on young adults from the Environmental Specimen Bank (ESB). Int. J. Hyg. Environ. Health.

[9] Lessmann F., Kolossa-Gehring M., Apel P., Rüther M., Pälmke C., Harth V., Brüning T., Koch H. (2019). German Environmental Specimen Bank: 24-hour urine samples from 1999 to 2017 reveal rapid increase in exposure to the para-phthalate plasticizer di(2-ethylhexyl) terephthalate (DEHTP). Environ. Int..

[10] Schmidtkunz C., Gries W., Weber T., Leng G., Kolossa-Gehring M. (2019). Internal exposure of young German adults to di(2-propylheptyl) phthalate (DPHP): Trends in 24-h urine samples from the German Environmental Specimen Bank 1999–2017. Int. J. Hyg. Environ. Health.

[11] Frederiksen H., Nielsen O., Koch H.M., Skakkebaek N.E., Juul A., Jørgensen N., Andersson A.M. (2020). Changes in urinary excretion of phthalates, phthalate substitutes, bisphenols and other polychlorinated and phenolic substances in young Danish men; 2009–2017. Int. J. Hyg. Environ. Health.

[12] Bastiaensen M., Gys C., Colles A., Malarvannan G., Verheyen V., Koppen G., Govarts E., Bruckers L., Morrens B., Franken C. (2021). Biomarkers of phthalates and alternative plasticizers in the Flemish Environment and Health Study (FLEHS IV): Time trends and exposure assessment. Environ. Pollut..

[13] Apel P., Kortenkamp A., Koch H.M., Vogel N., Rüther M., Kasper-Sonnenberg M., Conrad A., Brüning T., Kolossa-Gehring M. (2020). Time course of phthalate cumulative risks to male developmental health over a 27-year period: Biomonitoring samples of the German Environmental Specimen Bank. Environ. Int..

[14] Fréry N., Santonen T., Porras S.P., Fucic A., Leso V., Bousoumah R., Duca R.C., El Yamani M., Kolossa-Gehring M., Ndaw S. (2020). Biomonitoring of occupational exposure to phthalates: A systematic review. Int. J. Hyg. Environ. Health.

[15] Schwedler G., Rucic E., Lange R., Conrad A., Koch H.M., Pälmke C., Brüning T., Schulz C., Schmied-Tobies M.I.H., Daniels A. (2020). Phthalate metabolites in urine of children and adolescents in Germany. Human biomonitoring results of the German Environmental Survey GerES V, 2014–2017. Int. J. Hyg. Environ. Health.

[16] Lange R., Apel P., Rousselle C., Charles S., Sissoko F., Kolossa-Gehring M., Ougier E. (2021). The European Human Biomonitoring Initiative (HBM4EU): Human biomonitoring guidance values for selected phthalates and a substitute plasticizer. Int. J. Hyg. Environ. Health.

[17] Lemke N., Murawski A., Lange R., Weber T., Apel P., Dębiak M., Koch H.M., Kolossa-Gehring M. (2021). Substitutes mimic the exposure behaviour of REACH regulated phthalates—A review of the German HBM system on the example of plasticizers. Int. J. Hyg. Environ. Health.

[18] Barr D.B., Silva M.J., Kato K., Reidy J.A., Malek N.A., Hurtz D., Sadowski M., Needham L.L., Calafat A.M. (2003). Assessing human exposure to phthalates using monoesters and their oxidized metabolites as biomarkers. Environ. Health Perspect..

[19] Koch H.M., Gonzalez-Reche L.M., Angerer J. (2003). On-line clean-up by multidimensional liquid chromatography-electrospray ionization tandem mass spectrometry for high throughput quantification of primary and secondary phthalate metabolites in human urine. J. Chromatogr. B Analyt. Technol. Biomed Life Sci..

[20] Silva M.J., Samandar E., Preau JLJr Reidy J.A., Needham L.L., Calafat A.M. (2007). Quantification of 22 phthalate metabolites in human urine. J. Chromatogr. B Analyt. Technol. Biomed Life Sci..

[21] Schütze A., Pälmke C., Angerer J., Weiss T., Brüning T., Koch H.M. (2012). Quantification of biomarkers of environmental exposure to di(isononyl)cyclohexane-1,2-dicarboxylate (DINCH) in urine via HPLC-MS/MS. J. Chromatogr. B Analyt. Technol. Biomed Life Sci..

[22] Wittassek M., Angerer J., Kolossa-Gehring M., Schäfer S.D., Klockenbusch W., Dobler L., Günsel A.K., Müller A., Wiesmüller G.A. (2009). Fetal exposure to phthalates—A pilot study. Int. J. Hyg. Environ. Health.

[23] Koch H.M., Calafat A.M. (2009). Human body burdens of chemicals used in plastic manufacture. Philos. Trans. R. Soc. B Biol. Sci..

[24] Calafat A.M., Longnecker M.P., Koch H.M., Swan S.H., Hauser R., Goldman L.R., Lanphear B.P., Rudel R.A., Engel S.M., Teitelbaum S.L. (2015). Optimal Exposure Biomarkers for Nonpersistent Chemicals in Environmental Epidemiology. Environ. Health Perspect..

[25] Vorkamp K., Castaño A., Antignac J.-P., Boada L.D., Cequier E., Covaci A., Esteban López M., Haug L.S., Kasper-Sonnenberg M., Koch H.M. (2021). Biomarkers, matrices and analytical methods targeting human exposure to chemicals selected for a European human biomonitoring initiative. Environ. Int..

[26] Schindler B.K., Esteban M., Koch H.M., Castano A., Koslitz S., Canas A., Casteleyn L., Kolossa-Gehring M., Schwedler G., Schoeters G. (2014). The European COPHES/DEMOCOPHES project: Towards transnational comparability and reliability of human biomonitoring results. Int. J. Hyg. Environ. Health.

[27] Den Hond E., Govarts E., Willems H., Smolders R., Casteleyn L., Kolossa-Gehring M., Schwedler G., Seiwert M., Fiddicke U., Castaño A. (2015). First steps toward harmonized human biomonitoring in Europe: Demonstration project to perform human biomonitoring on a European scale. Environ. Health Perspect..

[28] Schantz M.M., Benner B.A., Heckert N.A., Sander L.C., Sharpless K.E., Vander Pol S.S., Vasquez Y., Villegas M., Wise S.A., Alwis K.U. (2015). Development of urine standard reference materials for metabolites of organic chemicals including polycyclic aromatic hydrocarbons, phthalates, phenols, parabens, and volatile organic compounds. Anal. Bioanal. Chem..

[29] Ganzleben C., Antignac J.P., Barouki R., Castaño A., Fiddicke U., Klánová J., Lebret E., Olea N., Sarigiannis D., Schoeters G.R. (2017). Human biomonitoring as a tool to support chemicals regulation in the European Union. Int. J. Hyg. Environ. Health.

[30] Esteban López M., Göen T., Mol H., Nübler S., Zarrabi K., Koch H.M., Dvorakova D., Hajslova J., Antignac J.P., Vacher V. (2021). The European Human Biomonitoring platform—Design and implementation of a QA/QC programme for selected priority chemicals. Int. J. Hyg. Environ. Health.

[31] Machtinger R., Gaskins A.J., Racowsky C., Mansur A., Adir M., Baccarelli A.A., Calafat A.M., Hauser R. (2018). Urinary concentrations of biomarkers of phthalates and phthalate alternatives and IVF outcomes. Environ. Int..

[32] Schwedler G., Conrad A., Rucic E., Koch H.M., Leng G., Schulz C., Schmied-Tobies M.I.H., Kolossa-Gehring M. (2020). Hexamoll^®^ DINCH and DPHP metabolites in urine of children and adolescents in Germany. Human biomonitoring results of the German Environmental Survey GerES V, 2014–2017. Int. J. Hyg. Environ. Health.

[33] (2015). Statistical Methods for Use in Proficiency Testing by Interlaboratory Comparison.

[34] Fearn T., Thompson M. (2001). A new test for ‘sufficient homogeneity’. Analyst.

[35] Thompson M., Ellison R., Wood R. (2006). The International Harmonized Protocol for the Proficiency Testing of Analytical Chemistry Laboratories. Pure Appl. Chem..

[36] Philippat C., Rolland M., Lyon-Caen S., Pin I., Sakhi A.K., Sabaredzovic A., Thomsen C., Slama R. (2021). Pre- and early post-natal exposure to phthalates and DINCH in a new type of mother-child cohort relying on within-subject pools of repeated urine samples. Environ. Pollut..

[37] Runkel A.A., Mazej D., Snoj Tratnik J., Tkalec Ž., Kosjek T., Horvat M. (2021). Exposure of men and lactating women to environmental phenols, phthalates, and DINCH. Chemosphere.

[38] Samandar E., Silva M.J., Reidy J.A., Needham L.L., Calafat A.M. (2009). Temporal stability of eight phthalate metabolites and their glucuronide conjugates in human urine. Environ. Res..

[39] Koch H.M., Schütze A., Pälmke C., Angerer J., Brüning T. (2013). Metabolism of the plasticizer and phthalate substitute diisononyl-cyclohexane-1,2-dicarboxylate (DINCH^®^) in humans after single oral doses. Arch. Toxicol..

[40] Blount B.C., Silva M.J., Caudill S.P., Needham L.L., Pirkle J.L., Sampson E.J., Lucier G.W., Jackson R.J., Brock J.W. (2000). Levels of seven urinary phthalate metabolites in a human reference population. Environ. Health Perspect..

[41] Koch H.M., Lessmann F., Swan S.H., Hauser R., Kolossa-Gehring M., Frederiksen H., Andersson A.-M., Thomsen C., Sakhi A.K., Bornehag G.C.-G. (2018). Analyzing terephthalate metabolites in human urine as biomarkers of exposure: Importance of selection of metabolites and deconjugation enzyme. J. Chromatogr. B.

