# Supplementary material for: Proficiency and Interlaboratory Variability in the Determination of Phthalate and DINCH Biomarkers in Human Urine: Results from the HBM4EU Project
Source: Toxics. 2022 Jan 26;10(2):57. doi: 10.3390/toxics10020057 (PMC8878211; doi:10.3390/toxics10020057)
Supplement: Supplementary file 1 [file toxics-10-00057-s001.zip › toxics-1511056-SI.pdf]

Supplementary Materials: Proficiency and Interlaboratory Variability in the Determination of Phthalate and DINCH Biomarkers in Human Urine: Results from the HBM4EU Project

Hans G. J. Mol, Ingrid Elbers, Claudia Pälmke, Daniel Bury, Thomas Göen, Marta Esteban López, Stefanie Nübler, Vincent Vaccher, Jean-Philippe Antignac, Darina Dvořáková, Jana Hajšlová, Amrit Kaur Sakhi, Cathrine Thomsen, Katrin Vorkamp, Argelia Castaño and Holger M. Koch

Table S1. Mean biomarkers concentration and overall RSD_r (N = 20) as determined during homogeneity assessment.

	Round-1				Round-2				Round-3				Round-4				Range tested	
	material R1A		material R1B		material R2A		material R2B		material R3A		material R3B		material R4A		material R4B		lowest	highest
Biomarker	ng/mL	RSD_r	ng/mL	RSD_r	ng/mL	RSDr	ng/mL	RSD_r	ng/mL	RSD_r	ng/mL	RSD_r	ng/mL	RSD_r	ng/mL	RSD_r	ng/mL	ng/mL
MEP	137	2%	138	1%	15.0	2%	40.2	2%	63.8	2%	100	4%	56.6	7%	137	2%	15.0	138
MBzP	2.17	3%	3.6	4%	0.869	5%	9.37	3%	< 0.2	-	2.26	4%	1.74	9%	2.33	4%	0.87	9.37
MiBP	7.94	2%	22.7	2%	9.00	2%	77.7	2%	1.03	12%*	14	6%	17	7%	17.5	2%	1.03	77.7
MnBP	11	3%	16.4	3%	6.56	3%	53.6	2%	1.05	13%	10.3	6%	13	8%	10.4	2%	1.05	53.6
MCHP	< 0.2	-	0.38	5%	< 0.2	-	0.98	5%	< 0.2	-	0.286	20%*	0.22	19%*	0.41	7%	0.220	0.980
MnPeP	< 0.2	-	1.43	3%	< 0.2	-	8.54	3%	< 0.2	-	1.09	10%	1.54	8%	2.24	4%	1.09	8.54
MEHP	1.58	8%	10.1	3%	< 0.5	-	5.39	3%	1.29	5%	4.82	4%	3.73	8%	4.47	2%	1.29	10.1
5OH-MEHP	10.7	2%	40.6	1%	4.28	1%	32.7	1%	2.74	5%	26.3	2%	11.9	7%	22.1	3%	2.74	40.6
5oxo-MEHP	5.35	2%	19.7	2%	1.75	3%	14.8	2%	1.48	7%	13.5	3%	5.55	8%	11.1	4%	1.48	19.7
5cx-MEPP	8.56	2%	36.5	3%	4.90	2%	32.4	1%	2.78	3%	29.2	3%	13.2	7%	21.9	1%	2.78	36.5
MnOP	1.18	3%	6.55	2%	0.215	5%	2.09	3%	0.296	7%	3.25	3%	1.24	7%	2.83	2%	0.215	6.55
OH-MiNP	7.46	3%	17.5	3%	1.67	3%	9.11	3%	1.13	5%	11.6	3%	5.56	6%	8.55	3%	1.13	17.5
cx-MiNP	7.35	3%	26.3	2%	1.67	6%	9.04	2%	1.6	6%	15.6	5%	7.29	7%	12.3	2%	1.60	26.3
OH-MiDP	6.9	2%	32	3%	3.1	3%	18.7	3%	1.71	4%	18.2	4%	10.7	7%	18.0	3%	1.71	32.0
cx-MiDP	5.28	3%	23.9	3%	1.68	3%	9.25	2%	1.92	6%	16	4%	7.96	6%	15.4	2%	1.68	23.9
OH-MINCH	3.28	1%	19.1	2%	6.91	2%	22.96	1%	1.11	2%	13.5	2%	11.5	3%	8.65	4%	1.11	23.0
cx-MINCH	3.16	3%	14.6	2%	3.53	2%	12.05	2%	1.17	2%	8.6	2%	7.51	4%	8.28	4%	1.17	14.6

* within-sample standard deviation (s_w) too high for adequate homogeneity assessment.

Table S2. Example sheets homogeneity assessment.

	Control material A		Control material A		Control material B		Control material B		Control material B	
	MBzP		cx-MiNP		MCHP		MnPeP		MEHP	
	replicate-1	replicate-2	replicate-1	replicate-2	replicate-1	replicate-2	replicate-1	replicate-2	replicate-1	replicate-2
1	0.82	0.89	1.92	1.76	0.20	0.35	1.18	1.17	4.96	4.78
2	0.89	0.91	1.71	1.78	0.31	0.21	0.94	1.03	4.87	4.62
3	0.84	0.91	1.84	1.71	0.34	0.29	1.22	1.20	5.01	4.90
4	0.80	0.83	1.69	1.70	0.37	0.30	1.22	0.89	5.16	4.91
5	0.87	0.88	1.64	1.66	0.32	0.34	1.09	0.98	4.90	4.79
6	0.94	0.88	1.66	1.72	0.31	0.37	1.08	1.11	4.65	4.94
7	0.88	0.91	1.64	1.66	0.23	0.23	1.25	1.02	4.63	4.82
8	0.92	0.84	1.51	1.66	0.25	0.28	1.17	0.96	4.93	4.92
9	0.83	0.80	1.46	1.61	0.21	0.31	1.09	1.11	4.78	4.43
10	0.86	0.87	1.50	1.65	0.20	0.29	0.97	1.10	4.85	4.59
grand mean	0.870		1.674		0.286		1.089		4.822	
Stdev	0.040		0.108		0.057		0.105		0.170	
VC%	5%		6%		20%		10%		4%	
Cochran's test										
C	0.277		0.214		0.358		0.445		0.249	
Ccrit	0.602		0.602		0.602		0.602		0.602	
C < Ccrit →	No outliers detected		No outliers detected		No outliers detected		No outliers detected		No outliers detected	
target σ_{FFP}	0.218		0.419		0.071		0.272		1.206	
s_x =	0.0326		0.0953		0.0406		0.0689		0.1291	
s_w =	0.0340		0.0773		0.0561		0.1106		0.1568	
s_s =	0.0220		0.0780		0.0086		0.0000		0.0661	
critical=$0.3\sigma_{FFP}$	0.0651		0.1256		0.0214		0.0817		0.3617	
s_s < critical?	Homogeneity adequate		Homogeneity adequate		Homogeneity adequate		Homogeneity adequate		Homogeneity adequate	
s_w< $0.5*\sigma_{FFP}$?	Method suited		Method suited		Method not suited		Method suited		Method suited	

Examples homogeneity assessment phthalate biomarkers from round-2 (left two) and round-3 (right three).

Table S2. (continued). Example sheets homogeneity assessment.

	Control material A OH-MINCH		Control material A cx-MINCH		Control material B OH-MINCH		Control material B cx-MINCH	
	replicate-1	replicate-2	replicate-1	replicate-2	replicate-1	replicate-2	replicate-1	replicate-2
1	6.64	6.83	3.51	3.57	23.34	23.30	12.31	12.46
2	6.91	7.04	3.53	3.62	23.25	23.12	11.92	12.43
3	6.83	6.90	3.51	3.63	23.05	22.81	12.28	12.20
4	6.71	6.76	3.49	3.38	23.00	22.56	12.31	12.10
5	6.79	6.92	3.51	3.51	23.00	23.04	12.21	11.95
6	6.91	7.12	3.54	3.57	22.26	22.55	11.81	12.06
7	6.94	7.03	3.43	3.56	23.47	23.10	11.81	12.16
8	7.03	7.06	3.47	3.63	22.31	23.20	11.97	11.65
9	6.95	6.93	3.52	3.46	22.70	23.03	11.87	11.72
10	6.87	7.09	3.58	3.53	23.17	22.89	11.98	11.76
grand mean	6.913		3.528		22.958		12.048	
Stdev	0.128		0.064		0.333		0.241	
VC%	2%		2%		1%		2%	
Cochran's test								
C	0.270		0.292		0.538		0.343	
Ccrit	0.602		0.602		0.602		0.602	
C < Ccrit →	No outliers detected		No outliers detected		No outliers detected		No outliers detected	
target σ_{FFP}	1.728		0.882		5.739		3.012	
s_x =	0.1111		0.0442		0.2762		0.2004	
s_w =	0.0947		0.0662		0.2713		0.1948	
s_s =	0.0887		0.0000		0.1988		0.1455	
critical=0.3σ_{FFP}	0.5185		0.2646		1.7218		0.9036	
s_s < critical?	Homogeneity adequate		Homogeneity adequate		Homogeneity adequate		Homogeneity adequate	
s_w< 0.5*σ_{FFP}?	Method suited		Method suited		Method suited		Method suited	

Examples homogeneity assessment DINCH biomarkers from round-2.

Table S3. Expert values used as assigned values in performance assessment rounds 2–4.

	Concentrations (mean of means) as derived from Expert Labs																	
	Urine R2A			Urine R2B			Urine R3A			Urine R3B			Urine R4A			Urine R4B		
Biomarker	ng/mL	*N*	u	ng/mL	*N*	u	ng/mL	*N*	u	ng/mL	*N*	u	ng/mL	*N*	u	ng/mL	*N*	u
MEP	17.39	5	5.1%	52.28	5	6.4%	71.77	5	3.5%	103.41	5	4.0%	51.48	4	3.5%	121.60	4	4.3%
MBzP	0.95	5	7.9%	10.36	5	4.8%	< 0.2			3.21	5	13.6%	2.03	4	6.6%	2.81	4	7.3%
MiBP	8.59	4	4.9%	69.88	4	7.1%	1.28	5	5.6%	15.32	5	2.9%	17.43	4	3.7%	17.22	4	3.5%
MnBP	6.64	5	4.7%	53.86	5	3.2%	1.03	5	7.6%	11.76	5	3.5%	13.95	4	2.2%	11.04	4	2.2%
MCHP	< 0.20	3		1.26	3	11.5%	< 0.2			0.45	3	**23.4%**	0.29	3	16.8%	0.53	3	12.5%
MnPeP	0.89	2		12.25	3	**21.0%**	*< 0.2*			1.32	4	9.4%	1.75	*3*	6.6%	2.50	3	5.6%
MEHP	0.57	3	9.6%	5.89	6	2.4%	1.21	5	16.5%	4.76	5	2.8%	3.33	4	4.4%	3.98	4	4.9%
5OH-MEHP	4.12	6	4.6%	32.29	6	4.3%	3.01	5	3.4%	27.10	5	3.1%	13.21	4	3.5%	23.23	4	2.0%
5oxo-MEHP	1.74	6	6.2%	14.56	6	6.3%	1.44	5	1.5%	12.86	5	2.2%	5.82	4	1.9%	11.10	4	1.9%
5cx-MEPP	5.41	6	3.6%	33.05	6	4.2%	3.22	5	5.9%	28.36	5	4.1%	15.64	4	7.0%	24.69	4	5.4%
MnOP	0.13	2		1.70	3	1.9%	0.36	3	**25.4%**	2.94	3	6.1%	1.36	4	16.4%	2.57	4	5.7%
OH-MiNP*	1.81	3	14.6%	11.19	3	15.3%	1.07	3	6.4%	8.83	3	**36.8%**	5.80	3	6.9%	8.17	3	2.4%
cx-MiNP*	2.64	4	13.8%	12.61	4	11.4%	2.04	5	7.9%	19.19	5	5.3%	9.25	4	8.4%	15.73	4	9.3%
OH-MiDP*	2.88	3	14.1%	17.17	3	11.2%	1.55	3	16.5%	19.13	3	3.7%	9.87	3	4.5%	15.93	3	6.5%
cx-MiDP*	1.95	3	12.3%	9.99	3	9.5%	1.80	5	7.6%	14.63	5	6.6%	7.19	4	3.8%	13.49	4	5.3%
OH-MINCH*	6.91	3	1.3%	22.95	3	0.9%	1.09	3	8.6%	12.99	3	2.0%	12.32	3	3.9%	9.71	3	5.8%
cx-MINCH*	3.66	3	2.3%	12.05	3	0.1%	1.09	3	3.4%	8.30	3	6.2%	7.07	3	10.6%	7.70	3	9.9%

N = number of expert-laboratories means used for determination of the expert values. u = relative uncertainty of the expert value (RSD divided by the square root of the number of expert laboratories).

* prescribed quantifier *m/z* transitions used: OH-MiNP 307 > 121; cx-MiNP 321 > 173; OH-MiDP 321 > 121; cx-MiDP 335 > 187; OH-MINCH 313 > 153; cx-MINCH 327 > 173

Values with grey background: expert value not suited and not used as assigned value because less than three expert laboratories involved or too high variability in expert laboratory results.

Table S4. List of expert laboratories in phthalate and DINCH biomarker QA/QC program.

COUNTRY	LABORATORY GROUP	INSTITUTION
Denmark (1,2)	Chemical Laboratory at Dep. of Growth and Reproduction	Rigshospitalet, Region Hovedstaden (RegionH)
Germany (1,2)	Institute for Prevention and Occupational Medicine of the German Social Accident Insurance (IPA)	Ruhr-Universität Bochum
Sweden (1*)	Occupational and environmental medicine	Lund University
USA (1)	Arizona Department of Health Services Laboratory	
USA (1,2)	Centers for Disease Control and Prevention (CDC)	
USA (1**)	New York State Department of Health, Wadsworth Center	

(1) Laboratory contributing as expert laboratory phthalate biomarkers (*R2 only, **R2&R3 only). (2) Laboratory contributing as expert laboratory DINCH biomarkers.

Table S5. Effect enzyme used for deconjugation on results of selected biomarkers.

		E.coli **based enzyme**				***H. Pomatia*** **based enzyme**				**Difference**	
**Sample**	**Biomarker**	**N**	**mean**	**SD**	**RSD**	**N**	**mean**	**SD**	**RSD**	**HP vs EC**	**t-test**
R2A	MEP	12	19.21	4.98	26%	3	23.24	6.33	27%	21%	not significant
R2A	MBzP	10	1.01	0.25	25%	3	1.26	0.42	34%	24%	not significant
R2A	MiBP	8	8.07	0.86	11%	3	10.93	0.80	7%	35%	**Significant**
R2A	MnBP	12	6.40	2.06	32%	3	9.50	0.87	9%	49%	**Significant**
R2A	MEHP	7	0.65	0.15	23%	4	1.43	0.37	26%	120%	**Significant**
R2A	5OH-MEHP	11	4.06	0.48	12%	6	4.15	1.31	32%	2%	not significant
R2A	5oxo-MEHP	11	1.68	0.24	15%	5	2.00	0.75	37%	19%	not significant
R2B	MEP	12	62.22	17.51	28%	3	61.20	8.83	14%	−2%	not significant
R2B	MBzP	11	10.50	2.22	21%	4	8.45	3.33	39%	−19%	not significant
R2B	MiBP	8	69.42	27.10	39%	4	63.09	13.73	22%	−9%	not significant
R2B	MnBP	12	54.38	12.46	23%	4	45.74	14.96	33%	−16%	not significant
R2B	MEHP	12	6.30	1.09	17%	5	6.45	1.37	21%	2%	not significant
R2B	5OH-MEHP	10	31.20	4.63	15%	6	25.63	8.15	32%	−18%	not significant
R2B	5oxo-MEHP	11	13.68	1.96	14%	6	11.90	3.62	30%	−13%	not significant

Data from control materials from round-2. Mean and SD are in ng/mL. Results were only used when the corresponding isotope analogue of the biomarker was used for quantification and when the number of results for both enzyme groups was at least three. Three example Box Whisker plots are shown in Figure S1.

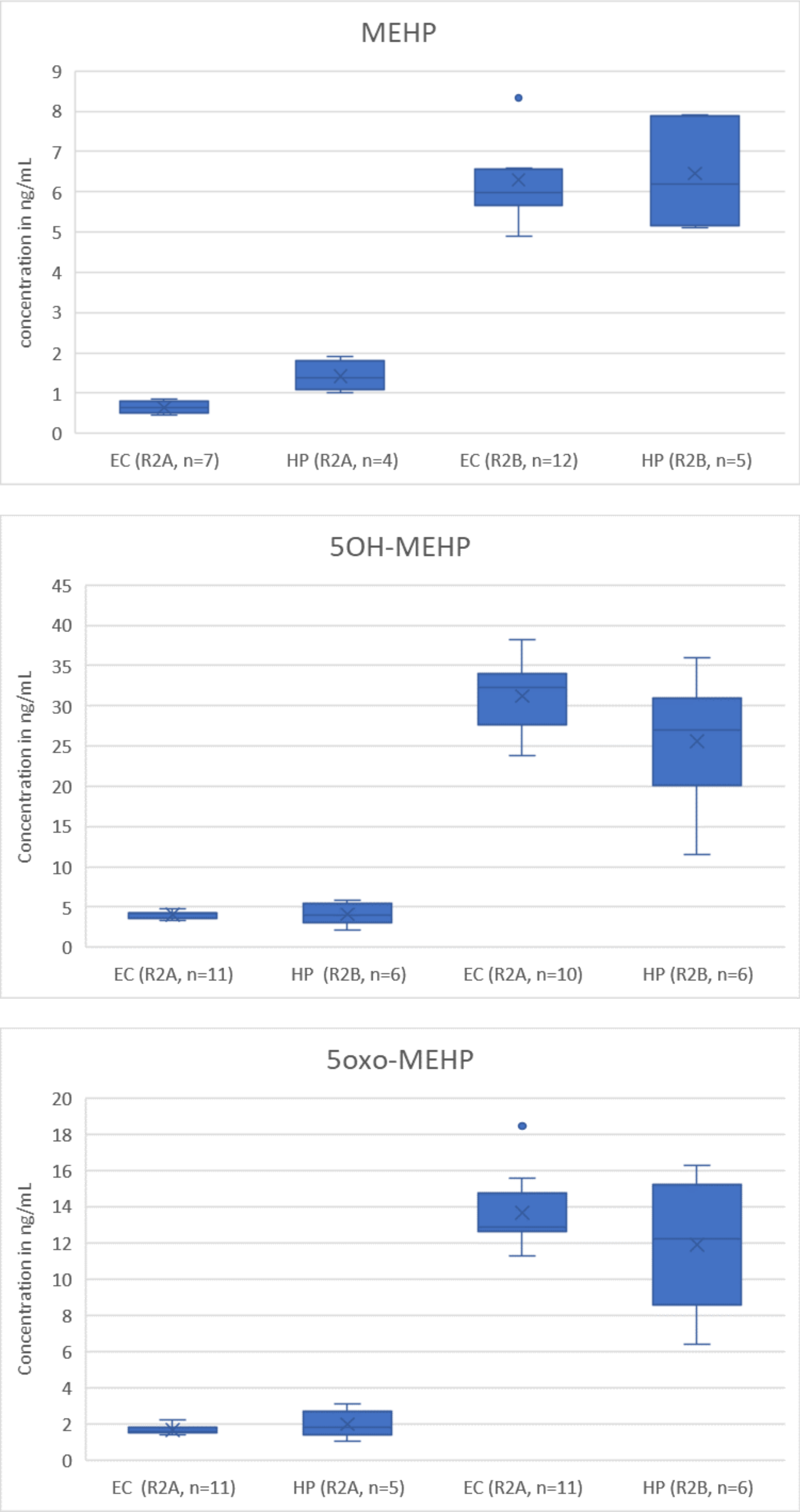

Figure S1. Effect enzyme used for deconjugation on results of selected biomarkers. Box-Whisker plots for results obtained for three example biomarkers in two control materials from round-2 (R2A and R2B) where either *E.coli* (EC) or *H. Pomatia* (HP) enzymes were used for deconjugation. Between brackets the number of results used. Other details see Table S5.

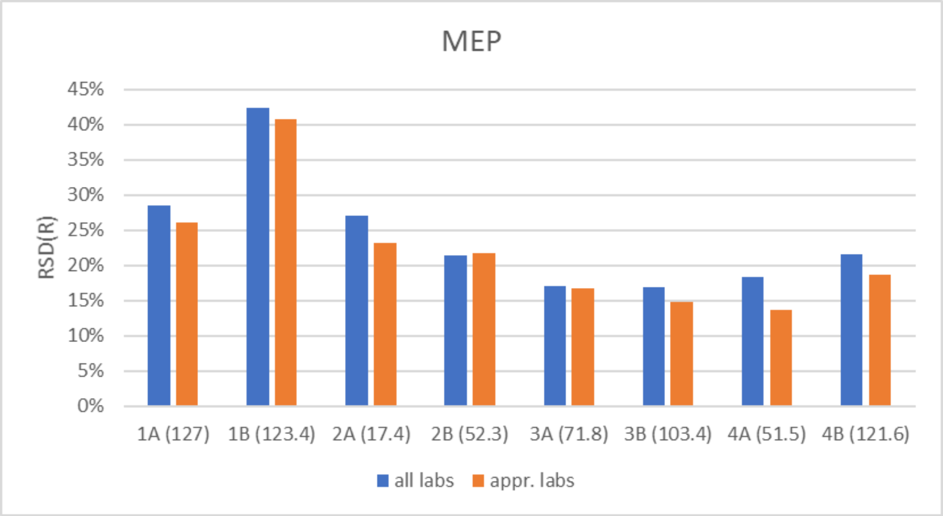

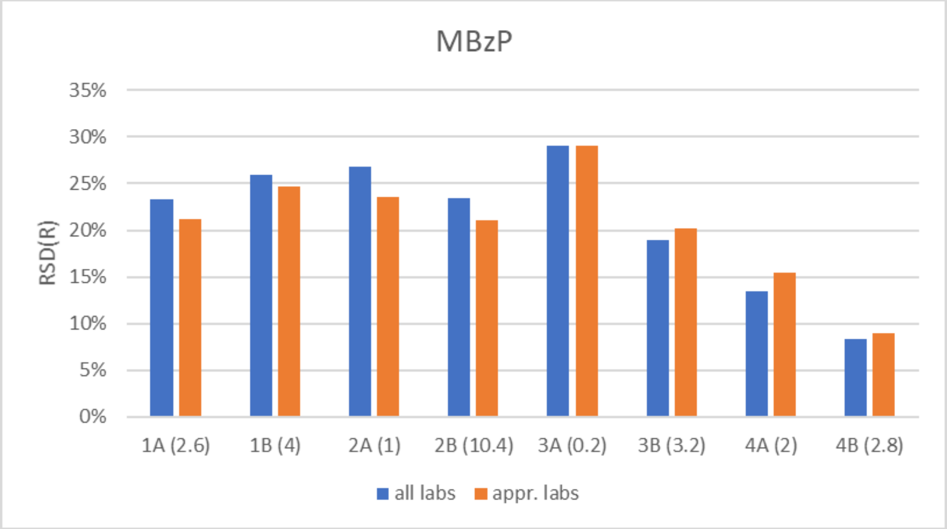

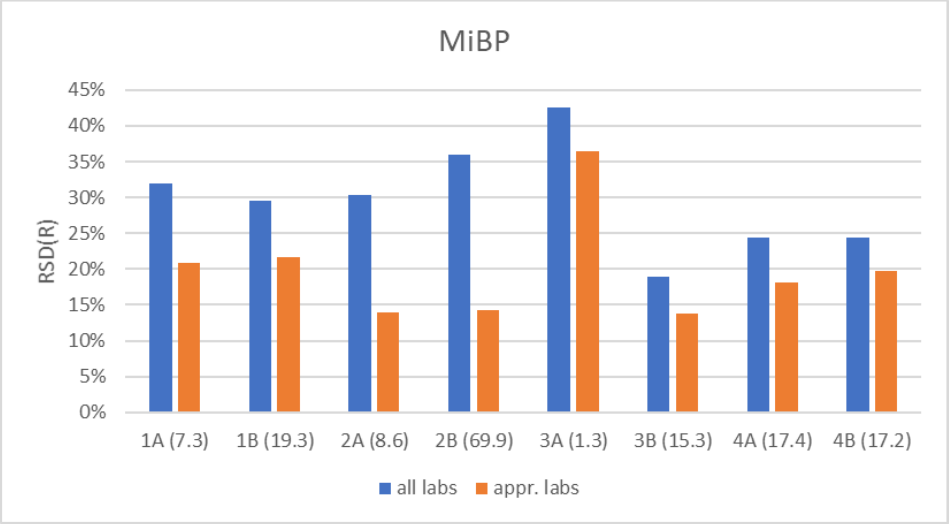

Figure S2. Interlaboratory variability of determination of phthalate and DINCH biomarkers in urine. RSD(R) = relative standard deviation, interlaboratory reproducibility. Legend X-axis: round/material number, between brackets the concentration of the biomarker in ng/mL. All labs = laboratories participating in the PT excluding the expert laboratories. Appr. labs = EU laboratories (participants and EU-based expert laboratories) approved for sample analysis within the frame of HBM4EU.

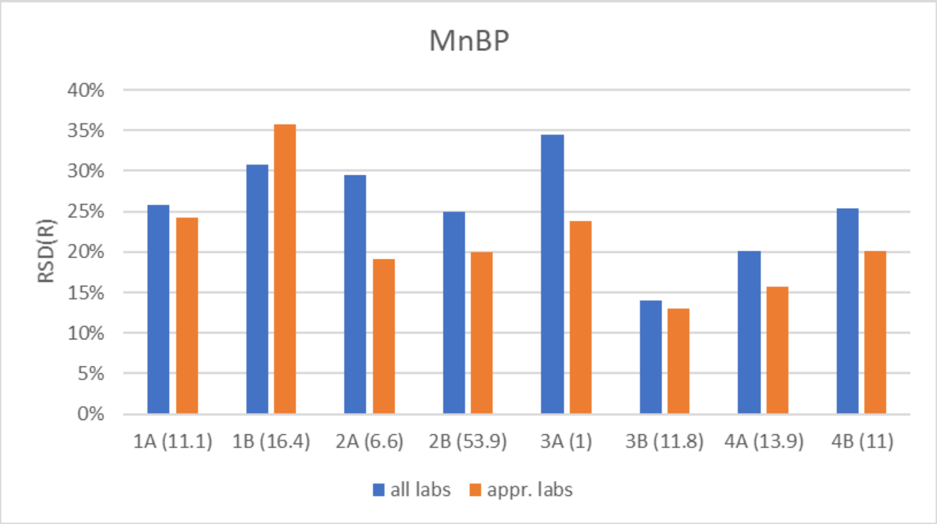

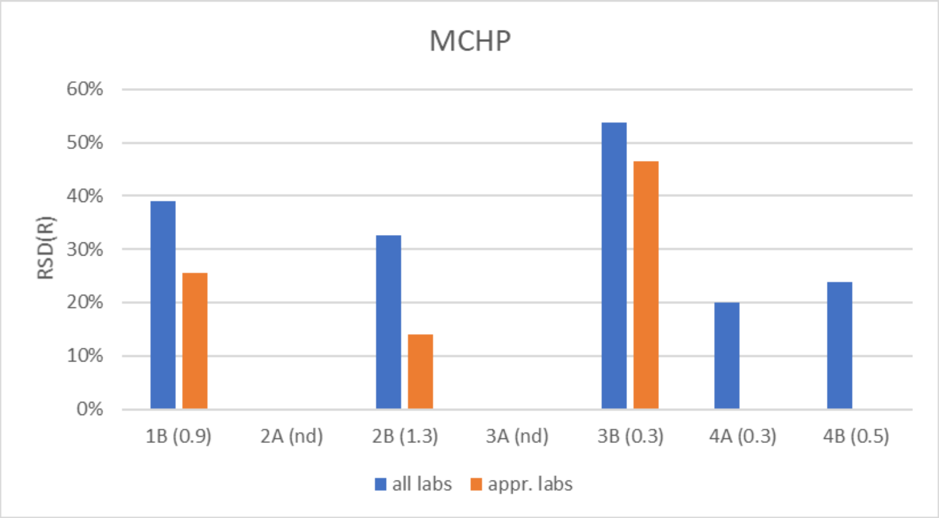

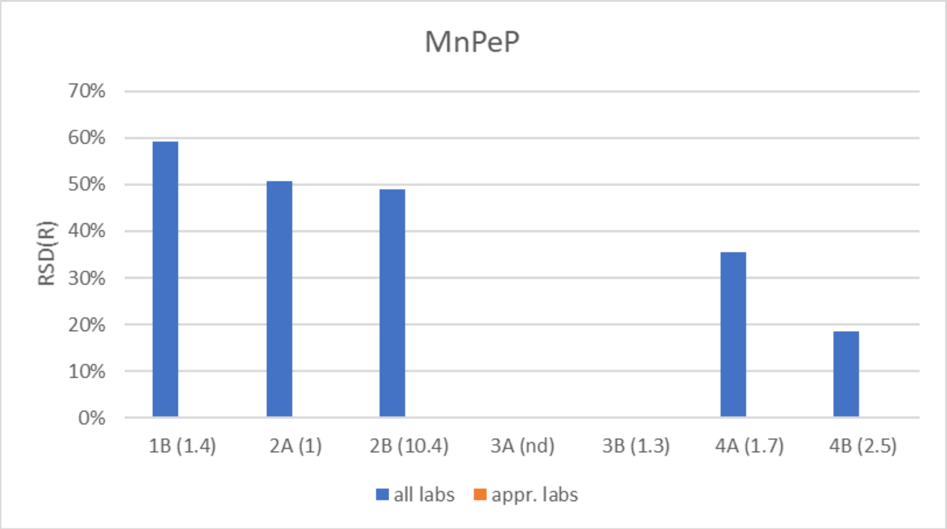

Figure S2. Interlaboratory variability of determination of phthalate and DINCH biomarkers in urine (continued). nd = not determined (biomarker not present). Appr. labs: RSD(R) not calculated because N < 7.

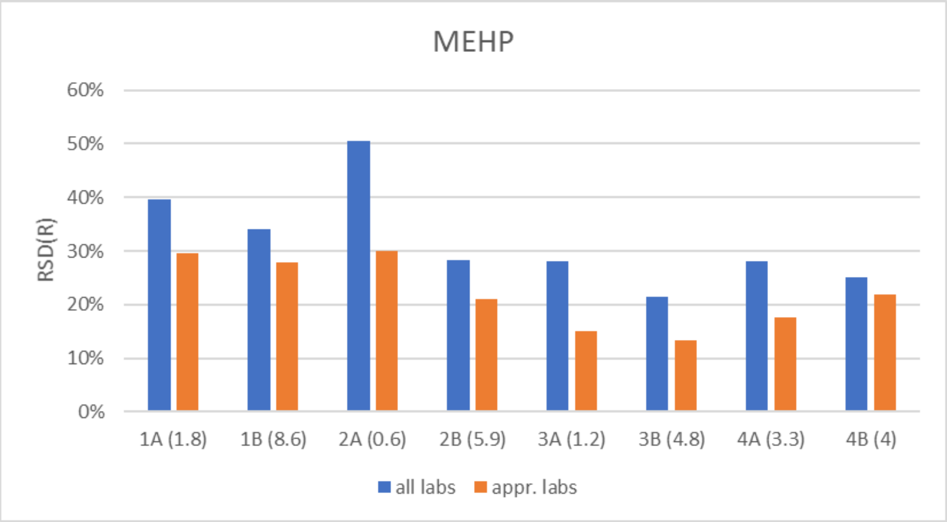

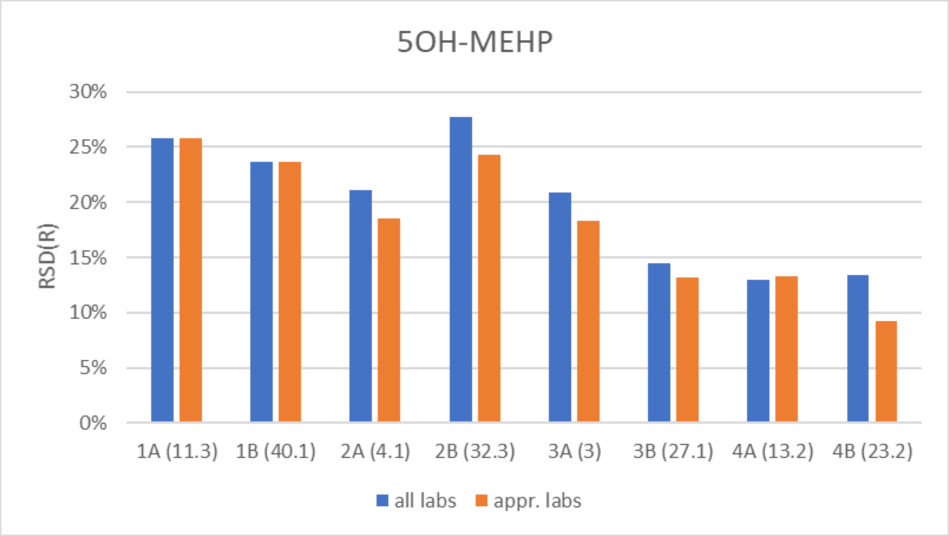

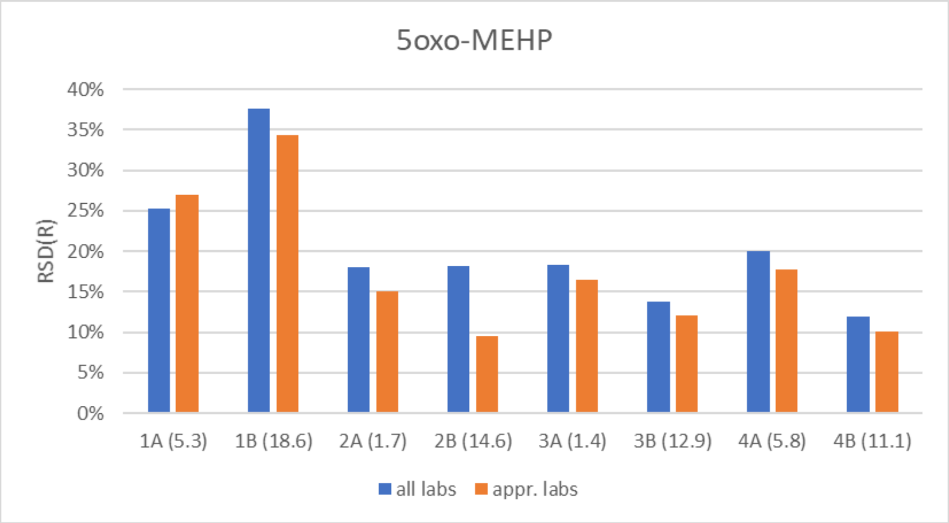

Figure S2. Interlaboratory variability of determination of phthalate and DINCH biomarkers in urine (continued).

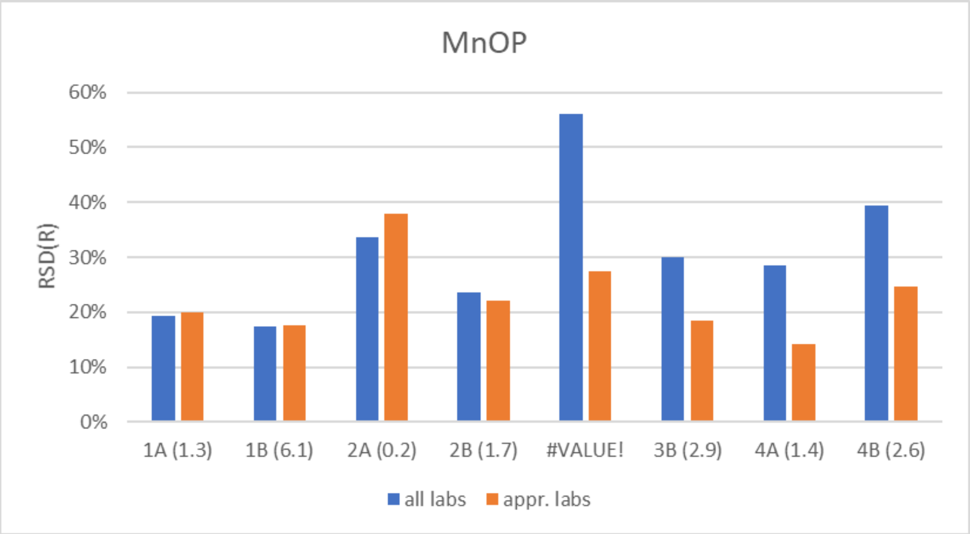

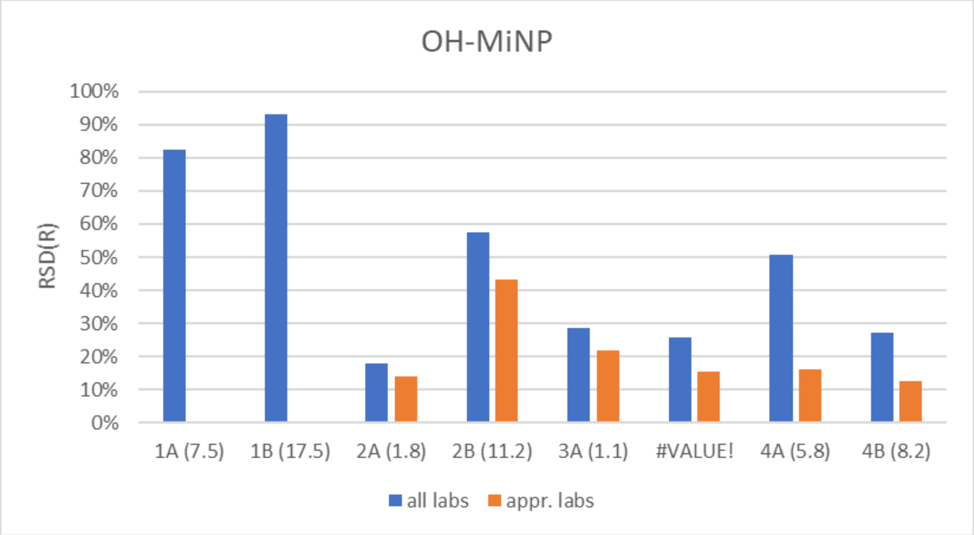

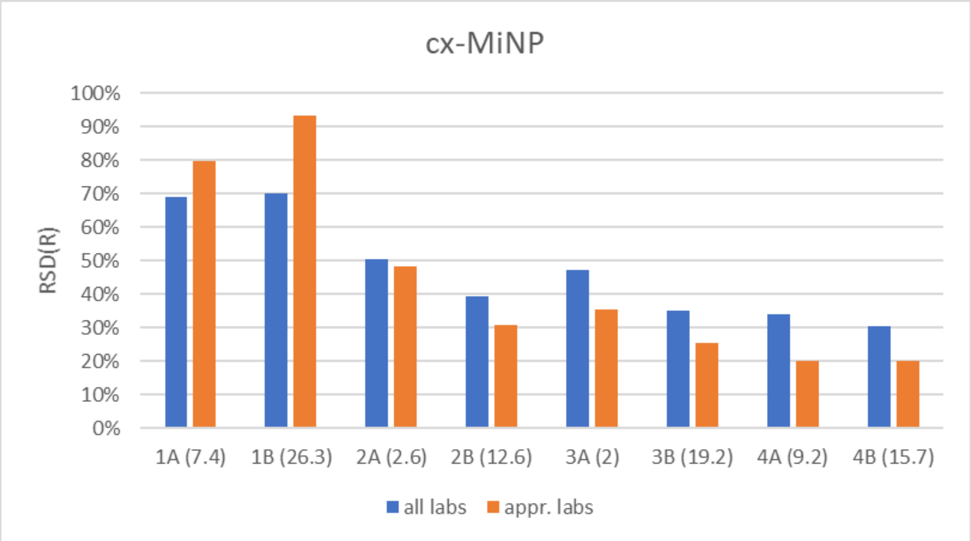

Figure S2. Interlaboratory variability of determination of phthalate and DINCH biomarkers in urine (continued). Appr. labs R1: RSD(R) not calculated because N < 7.

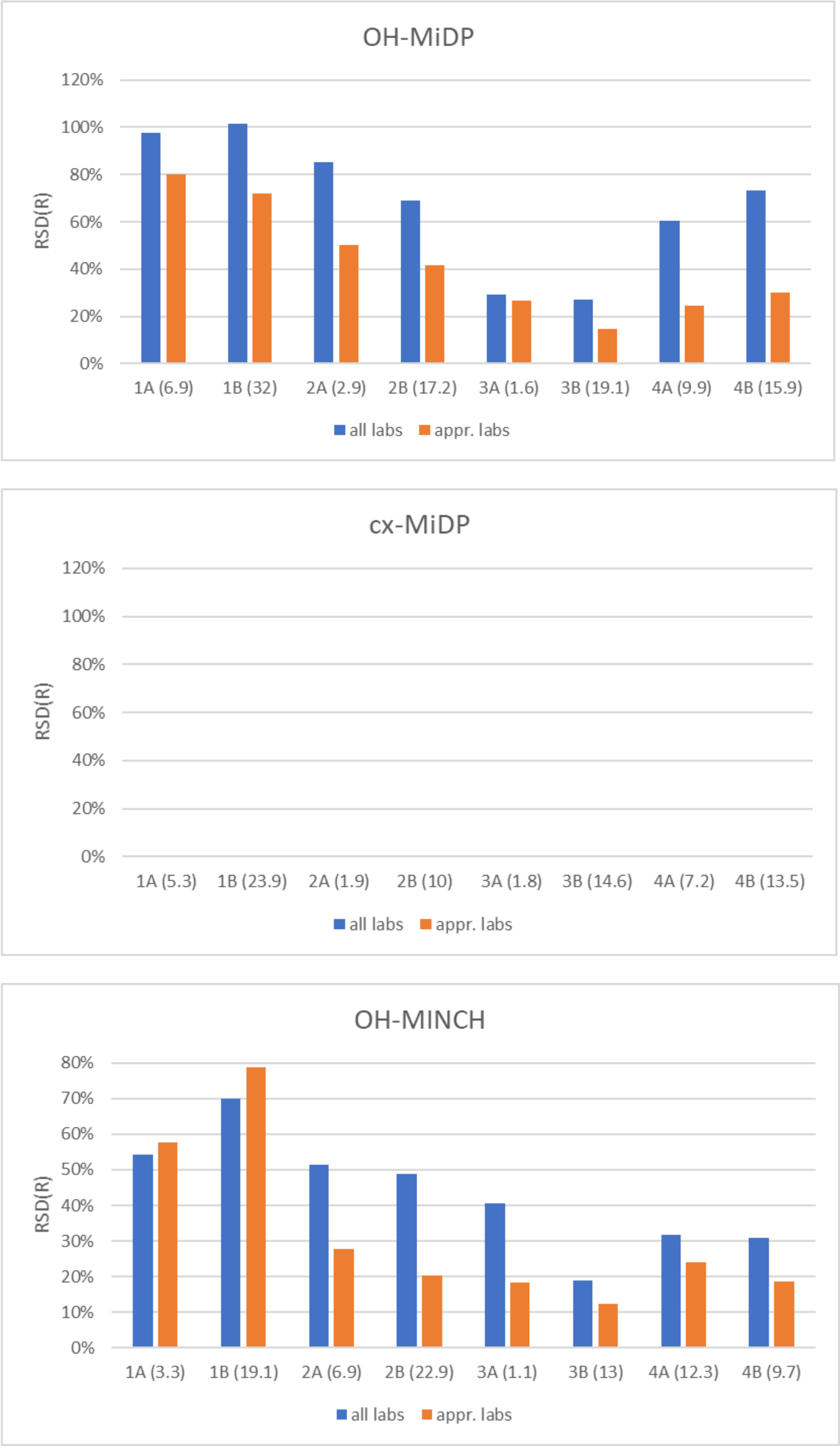

Figure S2. Interlaboratory variability of determination of phthalate and DINCH biomarkers in urine (continued). All labs and appr. labs: RSD(R) not calculated because N < 7.

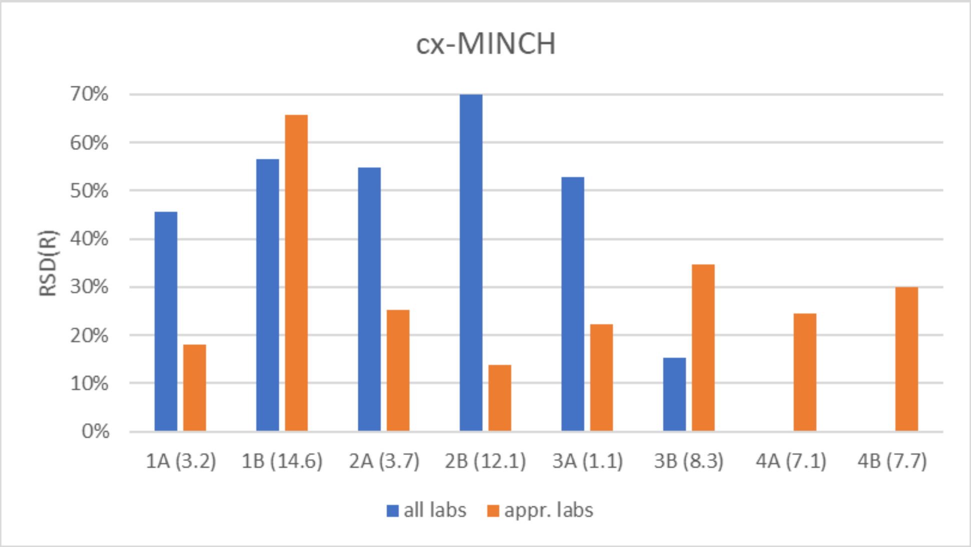

Figure S2. Interlaboratory variability of determination of phthalate and DINCH biomarkers in urine (continued). All labs: RSD(R) not calculated because N < 7. RSD(R) = relative standard deviation, interlaboratory reproducibility. Legend X-axis: round/material number, between brackets the concentration of the biomarker in ng/mL. All labs = laboratories participating in the PT excluding the expert laboratories. Appr. labs = EU laboratories (participants and EU-based expert laboratories) approved for sample analysis within the frame of HBM4EU.

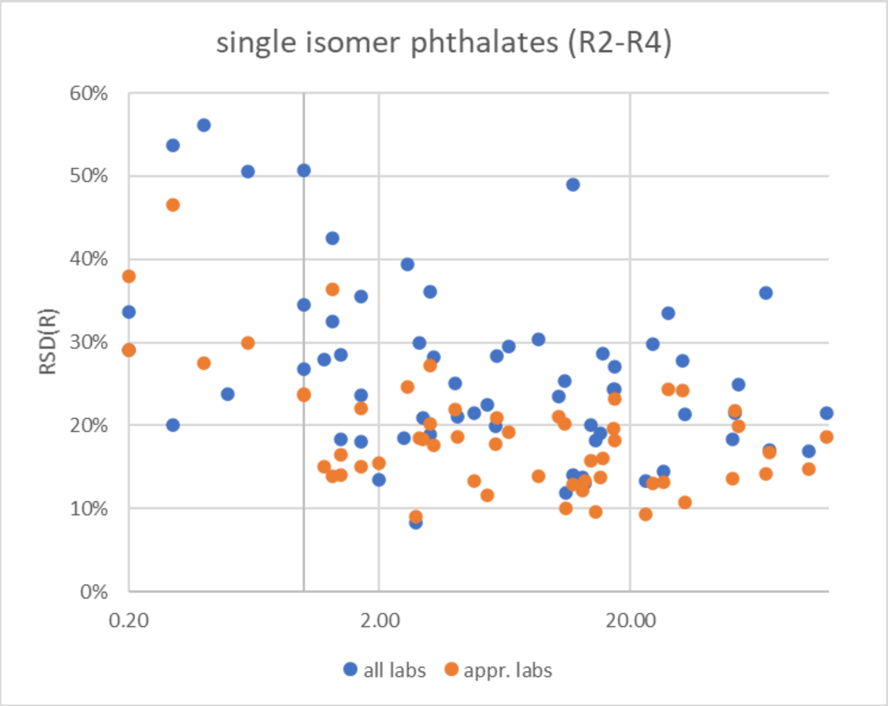

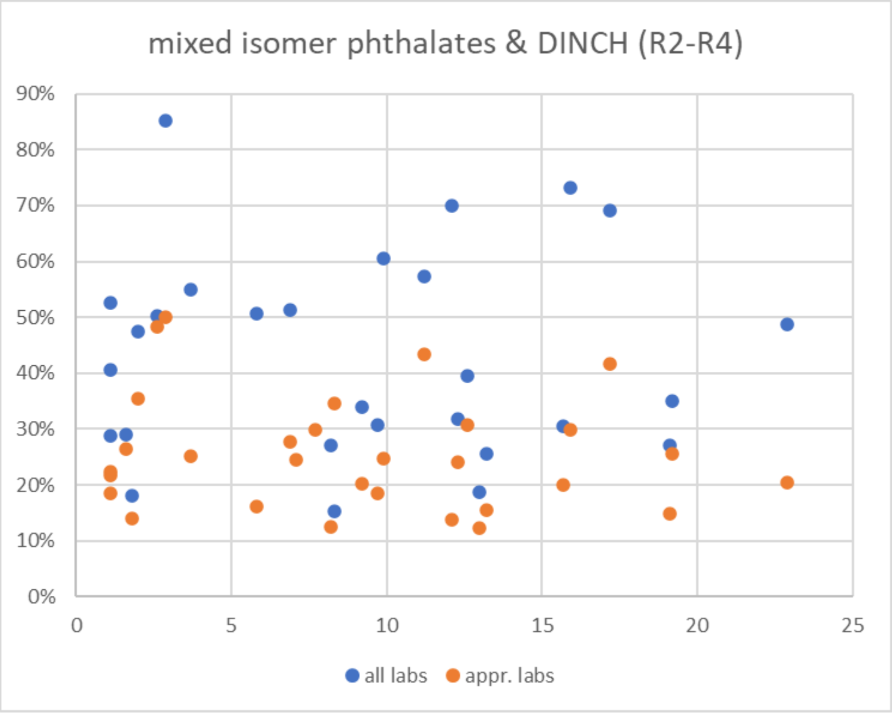

Figure S3. RSD_R (interlaboratory reproducibility) from rounds 2–4 versus concentration range of the biomarkers in urine. Top: average of 11 short-chain phthalate biomarkers. Note that the X-axis scale is logarithmic. Bottom: average of four long-chain phthalates and two DINCH biomarkers. Results shown for two groups of laboratories: all participants and laboratories approved in the frame of the HBM4EU project.
